# Wet Transfer of In Situ Grown Azo‐Containing Two‐Dimensional Conjugated Covalent Organic Framework Films for Photoswitchable Electronic Devices

**DOI:** 10.1002/anie.2260268

**Published:** 2026-06-07

**Authors:** Kexin Wang, Bin Han, Fan Qiu, Yeonsu Jeong, Yusheng Chen, Yubin Fu, Christos Gatsios, Marco Vittorio Nardi, Melanie Timpel, Yang Hou, Shun‐Qi Xu, Paolo Samorì

**Affiliations:** ^1^ University of Strasbourg & CNRS ISIS & icFRC, 8 Allée Gaspard Monge Strasbourg France; ^2^ Key Laboratory of Biomass Chemical Engineering of Ministry of Education College of Chemical and Biological Engineering, Zhejiang University Hangzhou China; ^3^ Institute of Quantum Science, Department of Physics Inha University Incheon South Korea; ^4^ College of Materials, and Fujian Key Laboratory of Surface and Interface Engineering for High Performance Materials Xiamen University Xiamen China; ^5^ IMEM‐CNR Institute of Materials for Electronics and Magnetism Trento Unit c/o Fondazione Bruno Kessler, Via alla Cascata 56/C Trento Italy; ^6^ School of Energy and Environment Southeast University Nanjing China

**Keywords:** covalent organic framework, field‐effect transistor, heterostructure, MoS_2_, photoswitch

## Abstract

Two‐dimensional π‐conjugated covalent organic frameworks (2D π‐COFs) combine in‐plane π‐delocalization with periodic open channels, making them versatile platforms for integrating functional groups into optoelectronic and neuromorphic systems. However, their predominant powder form has fundamentally constrained device integration and interfacial engineering. Herein, we demonstrate a general strategy for constructing high‐quality transition‐metal chalcogenide@2D π‐COF van der Waals heterostructures (vdWHs). Large‐area, atomically flat 2D π‐COF films are first grown on SiO_2_ substrates via a liquid–solid interfacial reaction and subsequently transferred onto monolayer MoS_2_ through a mild in situ wet‐transfer process. The resulting COF films exhibit atomic‐scale flatness and form clean interfaces, promoting efficient interfacial charge transfer in COF@MoS_2_ heterojunctions. As a model system, we employ a robust imidazole‐linked 2D π‐COF (COF‐Azo) functionalized with azobenzene moieties, whose reversible *trans‐cis* photoisomerization dynamically modulates the heterojunction's electronic structure. The COF‐Azo@MoS_2_ devices display pronounced and reversible photoswitching behavior, achieving a carrier density modulation of ∼6.7 × 10^11^ cm^−2^ and an on‐state current variation of ∼43.7%. Importantly, optical control over the isomerization fraction enables non‐volatile memory behavior within the vdWHs. This work establishes a scalable route to atomically flat 2D π‐COF films as viable building blocks for vdW heterostructures and introduces a robust platform for optically reconfigurable optoelectronics and memory devices.

## Graphical abstract

Wet transfer of an atomically flat azo‐functionalized 2D covalent organic framework film onto monolayer MoS_2_ enables clean van der Waals heterostructure devices with reversible photoswitching and multilevel nonvolatile memory.

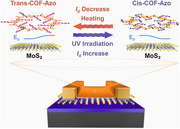

## 1 Introduction

Two‐dimensional π‐conjugated covalent organic frameworks (2D π‐COFs) are a class of crystalline conjugated porous polymers that are attracting ever‐increasing attention due to their programmable chemical structures, open‐channel architecture, extended in‐plane π‐conjugation, and tunable optoelectronic properties [1, 2]. These unique features render 2D π‐COFs ideal platforms for incorporating specific functional organic units into a 2D ordered skeleton, achieving a precise and robust assembly for target applications [3, 4, 5]. The most relevant examples include porphyrin‐based COFs for high‐performance photocatalysis [6, 7, 8], vinylene link‐lined COFs for solid state emission [9, 10], and nitrogen‐rich frameworks for energy storage [11, 12]. However, thus far, most 2D π‐COFs are synthesized under solvothermal [13, 14, 15] or ionothermal conditions [16, 17], typically yielding insoluble polycrystalline powders. As a result, although 2D π‐COFs possess appealing semiconducting properties, their integration into functional electronic devices remains extremely challenging [18]. Therefore, the development of methodologies to fabricate high‐quality COF films for application optoelectronic devices is highly sought after.

We recently reported a hybrid heterostructure obtained upon superimposing 2D COFs on molybdenum sulfide (MoS_2_), yielding COF@MoS_2_, thereby demonstrating the capability of 2D π‐COFs to modulate the semiconducting characteristics of MoS_2_ through interfacial electronic coupling [19]. In this respect, the development of photo‐switchable 2D COF films and their integration into a MoS_2_‐based heterostructure can represent a viable yet unexplored path to remotely light‐control electronic devices. Unfortunately, only a few photo‐switchable 2D π‐COFs have been synthesized to date, and most of them have been obtained as powders, preventing their incorporation into semiconducting devices [20, 21]. Most previously reported thin COF films have been prepared via interfacial synthesis at liquid/liquid or liquid/air interfaces [22, 23, 24, 25, 26, 27], which are generally limited to room temperature due to solvent evaporation, or via in situ growth on solid or liquid–solid interfaces at elevated temperatures, which has typically been believed to cause strong adhesion between the COF films and substrates [28, 29, 30], preventing straightforward transfer. Other approaches have also been explored, such as filtration‐assisted stacking of exfoliated 2D COF nanosheets [31] and mixed‐matrix membrane [32], which are mainly tailored for membrane‐based separation applications and are not inherently designed for direct integration into electronic device architectures. In this context, establishing a methodology to construct transferable 2D π‐COF films at higher temperature can significantly broaden the family of 2D π‐COF films and enable their application in remotely controlled functional optoelectronic devices.

**FIGURE 1:**
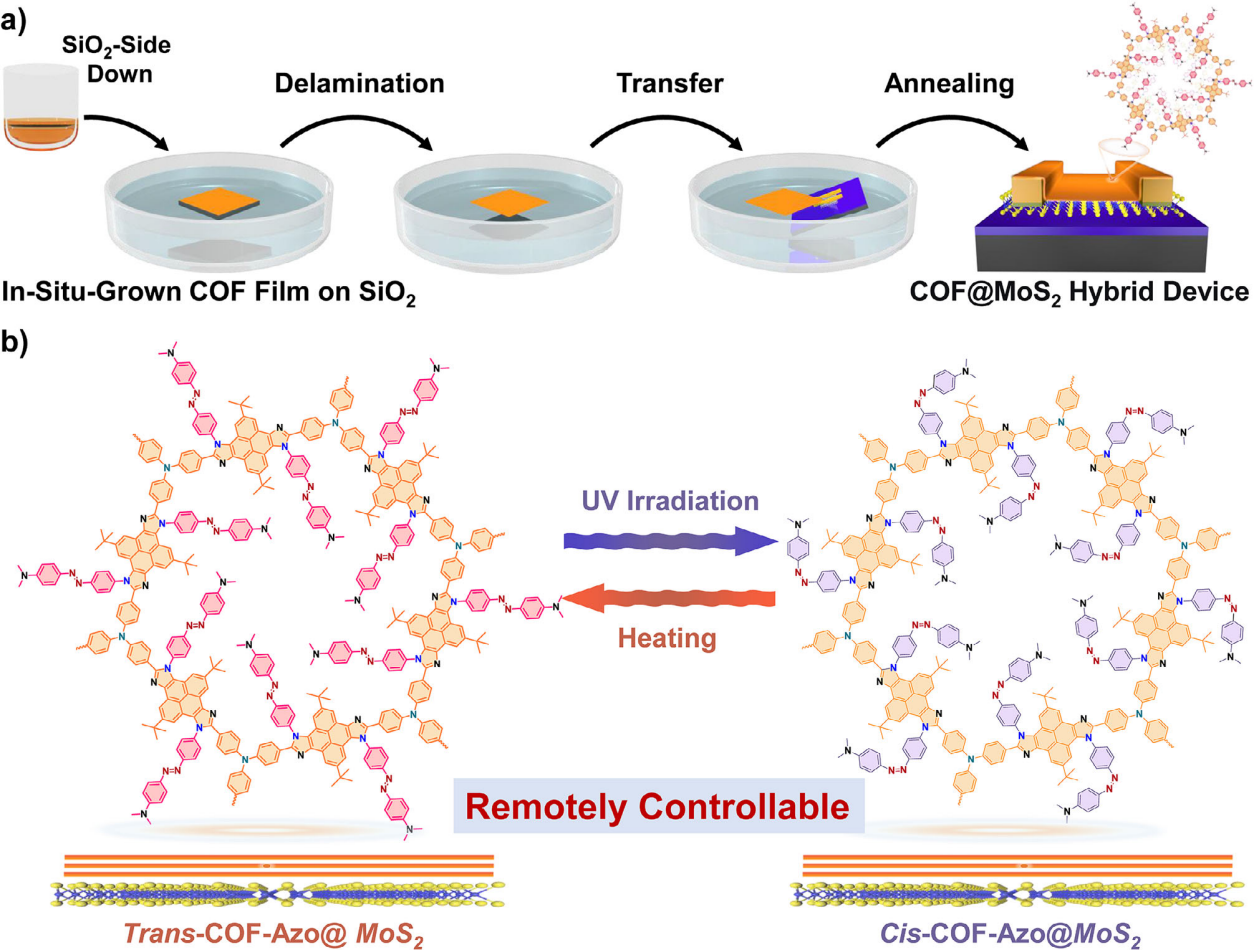
Schematic illustration of device fabrication and photo‐responsive mechanism. a) Wet‐transfer‐assisted fabrication of COF@MoS_2_ hybrid device; b) Mechanism of the photo‐responsive behavior, in which blue and yellow spheres correspond to Mo and S atoms.

In this work, we demonstrate a “wet transfer of in situ grown COF films” strategy to fabricate a robust azobenzene‐containing atomically flat 2D π‐COF (COF‐Azo) film on monolayer MoS_2_ flakes for constructing photo‐switchable semiconducting devices (Figure 1a). COF‐Azo was first synthesized under solvothermal conditions, which was applied for its in situ growth on a sacrificial SiO_2_ substrates via a liquid–solid interfacial reaction. Benefiting from the atomically flat surface nature of SiO_2_, a continuous film with an ultralow surface roughness (∼0.47 nm) on the contact side was achieved. Interestingly, the high‐quality 2D π‐COF films can be gently wet‐transferred onto a monolayer MoS_2_ field‐effect transistor (FET) channels [33, 34], forming COF‐Azo@MoS_2_ hybrid van der Waals heterojunctions (vdWHs). Notably, the COF‐Azo@MoS_2_ hybrid vdWHs exhibited a switchable and light‐controlled charge transport behavior, arising from *trans‐cis* isomerization of the azobenzene side chains (Figure 1b), which induced a carrier concentration modulation up to ∼6.71 × 10^11^ cm^−2^ along with an increase of the on‐state current by ∼43.7%. Moreover, as a proof‐of‐concept, controlled partial isomerization further enables non‐volatile multi‐level memory states, demonstrating remote and programmable tuning of the electronic output. This work introduces a versatile strategy for fabricating 2D π‐COF films and highlights the great potential of the 2D COF@MoS_2_ hybrid systems for light‐responsive neuromorphic applications.

## 2 Results and Discussion

### 2.1 Synthesis and Characterization of COF‐Azo

**FIGURE 2:**
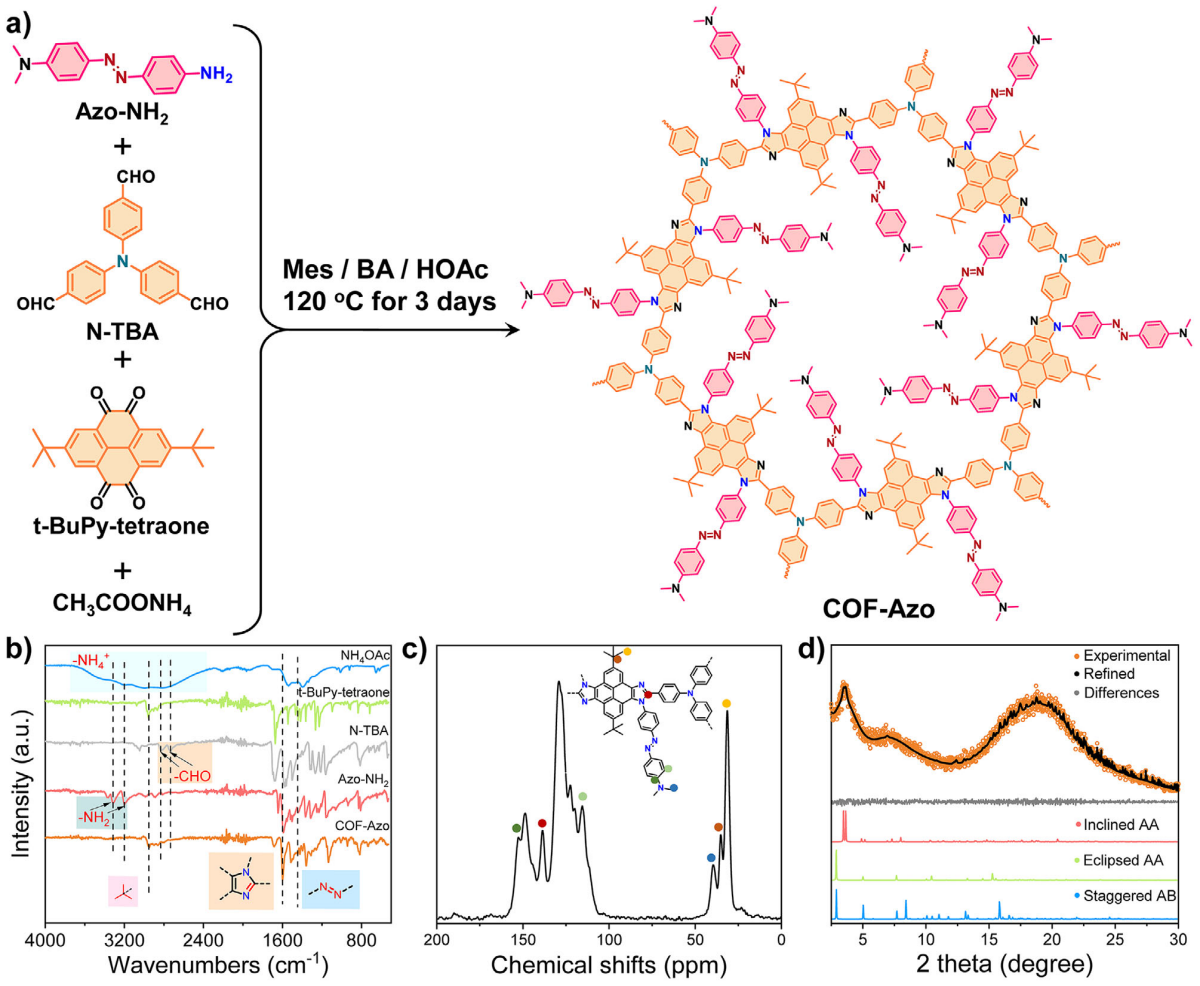
Synthesis and characterization of COF‐Azo. a) Schematic illustration of the synthesis of COF‐Azo; b) FT‐IR spectra of COF‐Azo and its corresponding monomers; c) Solid‐state ^13^C MAS‐NMR spectrum of COF‐Azo; d) Experimental and simulated PXRD patterns of COF‐Azo. The orange, black, gray, red, green, and blue traces correspond to the experimental, simulated, difference, inclined AA, eclipsed AA, and staggered AB stacking models.

COF‐Azo was first synthesized by heating 4,4′,4″‐nitrilotribenzaldehyde (N‐TBA), *N,N*‐dimethyl‐4,4‐azodianiline (Azo‐NH_2_), 2,7‐ditert‐butylpyrene‐4,5,9,10‐tetraone (t‐BuPy‐tetraone), and ammonium acetate (CH_3_COONH_4_) in a mixed solvent of mesitylene (Mes) and benzyl alcohol (BA) with the acetic acid (HOAc, 6 M) as catalyst at 120°C for 3 days (Figure 2a).

The chemical structure of COF‐Azo was confirmed by Fourier transform infrared (FT‐IR) and solid‐state ^13^C magic angle spinning nuclear magnetic resonance (MAS‐NMR) spectra. The efficient reaction occurrence was evidenced by FT‐IR spectroscopy of COF‐Azo (Figure 2b), revealing the disappearance of the signals at 3400–3200 cm^−1^ (─NH_2_), 2830 and 2737 cm^−1^ (─CHO), and the broad peaks at ∼3000 (─NH_4_^+^), while new peaks at 1601 cm^−1^ (─C═N─ in imidazole), 1442 cm^−1^ (─N═N─ of azobenzene), and 2958 cm^−1^ (tertbutyl groups attached to pyrene) appeared. In the solid‐state ^13^C MAS‐NMR spectrum of COF‐Azo (Figure 2c), the signals at 39.5, 35.2, and 31.4 ppm correspond to the tert‐butyl and methyl groups, while the peak at 138.6 ppm can be assigned to C═N in imidazole. In particular, the attached methyl groups (*N,N*‐methyl groups in Azo‐NH_2_) signal at 39.5 ppm provides unambiguous evidence for the successful integration of the azobenzene moieties. Therefore, both the FT‐IR and solid‐state ^13^C MAS‐NMR spectra validated the successful imidazole polymerization and the incorporation of azobenzene moieties into the COF‐Azo framework.

The crystalline structure of COF‐Azo was elucidated by powder xX‐ray diffraction (PXRD) and high‐resolution transmission electron microscopy (HR‐TEM). Figure 2d shows that the experimental PXRD pattern was characterized by diffraction peaks at approximately 3.67°, 7.12°, and 18.15°, which can be attributed to the (100), (200), and (001) planes, respectively. It should be mentioned that the observed moderate crystallinity originates from the steric hindrance of the tert‐butyl substituents, which were deliberately incorporated to attenuate excessive interlayer π–π stacking and create the spatial freedom required for efficient *trans‐cis* isomerization within the framework. In addition, the comparison between the PXRD profiles of the monomeric precursors and the COF‐Azo (Figure S1) unambiguously confirmed the formation of a new crystalline phase, indicating a complete polymerization. Subsequently, three possible stacking configurations with eclipsed AA, staggered AB, and inclined AA stacking were built to study the stacking configurations, using Materials Studio Software (Figures S2–S4). The simulated PXRD patterns of incline AA stacking model matched well with the experimental data. Further Pawley refinement based on the inclined AA stacking model gave the unit cell parameters of *a* = 37.2 Å, *b* = 31.9 Å and *c* = 8.0 Å, *α* = 49.9° *β* = 136.7° and *γ* = 119.8°, with refinement factors of *R*_wp_ = 5.16% and *R*_p_ = 4.10%.

The HR‐TEM image (Figure S5) revealed lattice fringes with an interplanar spacing of 0.45 nm, corresponding to the (001) plane. The pore size of COF‐Azo is too small to be reliably assessed by N_2_ adsorption–desorption measurements, likely due to the inclined stacking mode and the tert‐butyl groups. [35, 36] Nevertheless, the moderate crystallinity arising from steric hindrance of the tert‐butyl groups does not impair the overall porous architecture, but rather favors the *trans‐cis* photoisomerization of the azobenzene moieties. Notably, CO_2_ adsorption–desorption measurements confirm the presence of microporosity, with a surface area of 111.07 m^2^ g^−1^ (at *P*/*P*_0_ = 0.03) and pore size distributions at the range of 0.45–0.87 nm (Figure S6). Thermogravimetric and differential scanning calorimetry (TGA/DSC) analyses (Figure S7) highlighted the superior thermal robustness of COF‐Azo. When compared to the corresponding azobenzene monomer, COF‐Azo displayed significantly improved thermal stability (~300°C vs. ~200°C), which can be ascribed to the extended π‐conjugation and rigid covalent network.

### 2.2 Characterization of COF‐Azo Film and Hybrid System

The COF‐Azo film was assembled following the procedure illustrated in Figure 1a. During the solvothermal synthesis, the SiO_2_ substrate was immersed in the reaction mixture with its smooth side facing downward to prevent aggregate formation, allowing most particles to settle by gravity. Under these conditions, a thin COF film gradually formed onto the SiO_2_ surface, while polycrystalline COF powders simultaneously precipitated at the bottom of the reaction tubes. The as‐grown COF films on the SiO_2_ were sequentially washed by immersion into dimethylformamide (DMF), tetrahydrofuran (THF), and acetone, for 2 h each, to remove unreacted molecules and oligomers. Afterward, a polystyrene (PS) solution was spin‐coated onto the COF film, which was then floated on the surface of distilled water. Within the timescale of ∼30 min, the PS‐coated COF film remained floating on the water surface, whereas the SiO_2_ substrates detached and sank, yielding a freestanding COF‐Azo film supported by a PS layer.

**FIGURE 3:**
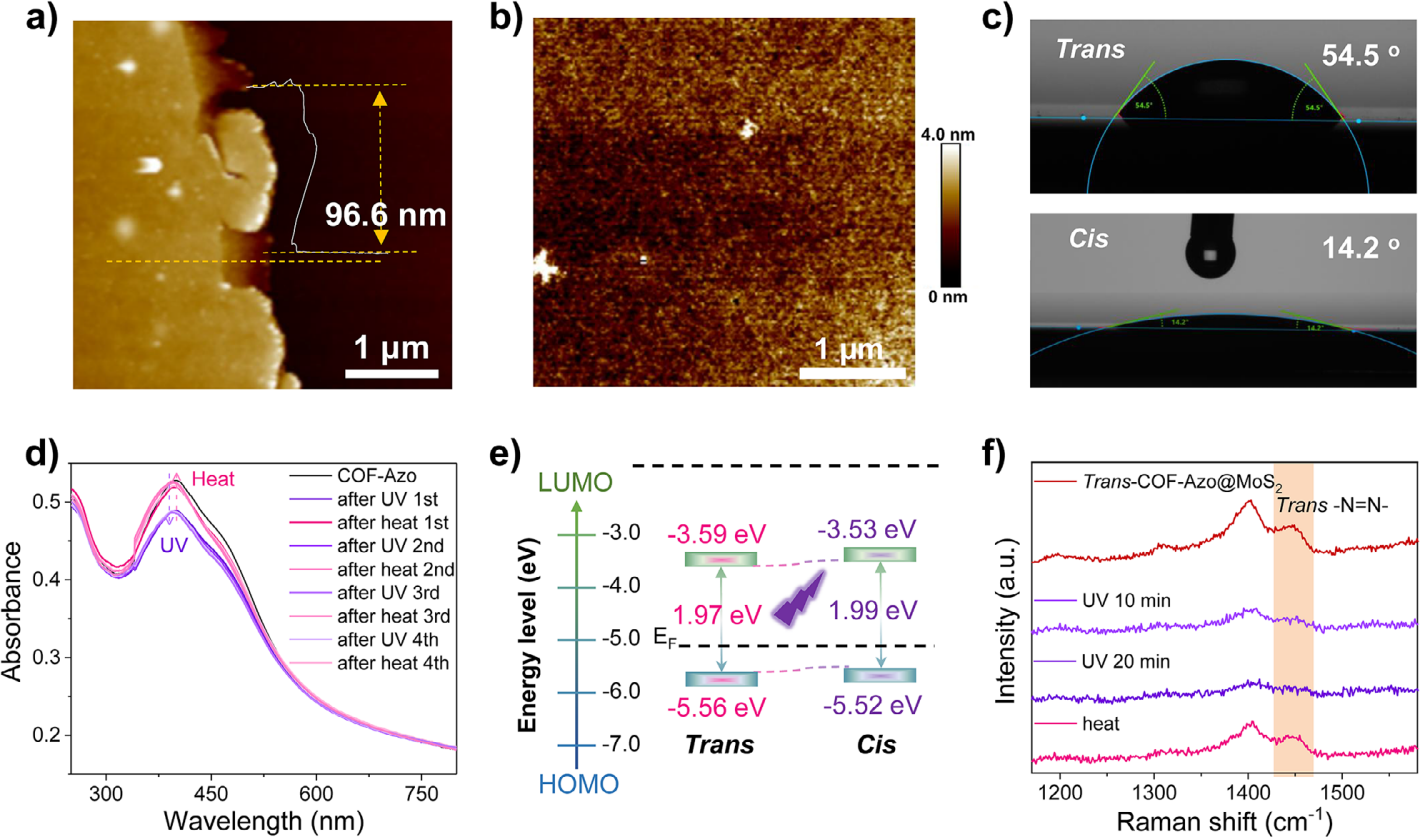
Characterization of COF‐Azo film. a) AFM image of COF‐Azo film used for thickness measurements; b) AFM image of COF‐Azo film used for roughness measurements; c) Water contact angle images of *trans‐* and *cis‐* COF‐Azo films; d) UV–vis absorption spectra of COF‐Azo films under alternating UV irradiation and thermal treatment; e) Schematic illustration of the HOMO–LUMO energy levels and optical bandgap evolution for *trans‐* and *cis‐* COF‐Azo films; f) Raman spectra of the COF‐Azo@MoS_2_ hybrid device under UV and thermal conditions.

Atomic force microscopy (AFM) measurements revealed a uniform thickness of ∼96.6 nm for the COF‐Azo film after removal of the PS (Figure 3a). Remarkably, benefiting from the in situ growth on the atomically smooth SiO_2_ substrate acting as template, the COF‐Azo film exhibited an ultraflat bottom interface with a root mean square roughness (*R*_RMS_) as low as ∼0.47 nm as determined on a region of 3 × 3 µm^2^ (Figure 3b), which is essential for achieving high‐quality van der Waals contact with MoS_2_. In contrast, the COF‐Azo upper film's surface showed a much higher roughness, which because of its magnitude could not be assessed by an AFM but rather with profilometry (Figure S8), underscoring the pronounced templating effect of the SiO_2_ substrates. As shown in Figure S9, the FT‐IR spectrum of the COF‐Azo film displayed characteristic vibrational bands identical to those observed in the bulk material, confirming the retention of the framework's chemical integrity upon thin‐film formation.

The light‐responsive characteristics of the COF‐Azo film, arising from the reversible *trans‐cis* photo‐isomerization of azobenzene units (termed as *trans‐*COF‐Azo and *cis*‐COF‐Azo), were further investigated. Water contact angle measurements revealed that the *cis*‐COF‐Azo was significantly more hydrophilic (contact angle = 14.2°) than that of *trans*‐COF‐Azo (contact angle = 54.5°), indicating an efficient isomerization (Figure 3c). As shown in Figure 3d, the azobenzenes in the COF scaffold could be reversibly toggled between the *trans*‐ and *cis*‐ states through alternating UV irradiation and thermal treatment, with high reversibility demonstrated over four switching cycles. Notably, the optical response intensity displayed a stepwise variation with prolonged irradiation or heating intervals (Figure S10), suggesting the controlled isomerization kinetics within the COF framework.

The frontier orbital energy levels of the COF‐Azo films were determined by cyclic voltammetry (CV) and ultraviolet–visible (UV–vis) absorption spectra. As shown in Figures 3e and S11, the highest occupied molecular orbital (HOMO) energy levels of the *trans*‐COF‐Azo and *cis*‐COF‐Azo films were measured to be −5.56 and −5.52 eV, respectively. The optical band gaps, estimated from the UV–vis absorption spectra, resulted ∼1.97 eV for *trans*‐COF‐Azo and ∼1.99 eV for *cis*‐COF‐Azo. The corresponding lowest unoccupied molecular orbital (LUMO) energy levels were calculated to be −3.59 and −3.53 eV, respectively. These results clearly indicate that the *trans‐cis* isomerization can be exploited to effectively modulate the optoelectronic characteristics of the COF films.

Encouraged by the smooth surface morphology and photoswitchable characteristics, the COF‐Azo film was transferred onto MoS_2_‐based semiconducting devices to evaluate its photoisomerization behavior under operational conditions. The characteristic vibrational band at 1450 cm^−1^, corresponding to the *trans‐*azobenzene configuration, gradually diminished under UV illumination and reappeared upon thermal treatment, evidencing a reversible and controllable in situ photoisomerization process within the COF structure on the COF@MoS_2_ hybrid device (Figure 3f).

### 2.3 Electrical Characterization of Photo‐Responsive COF‐Azo@MoS_2_ vdW Heterostructures and Application for Non‐volatile Memory Devices

**FIGURE 4:**
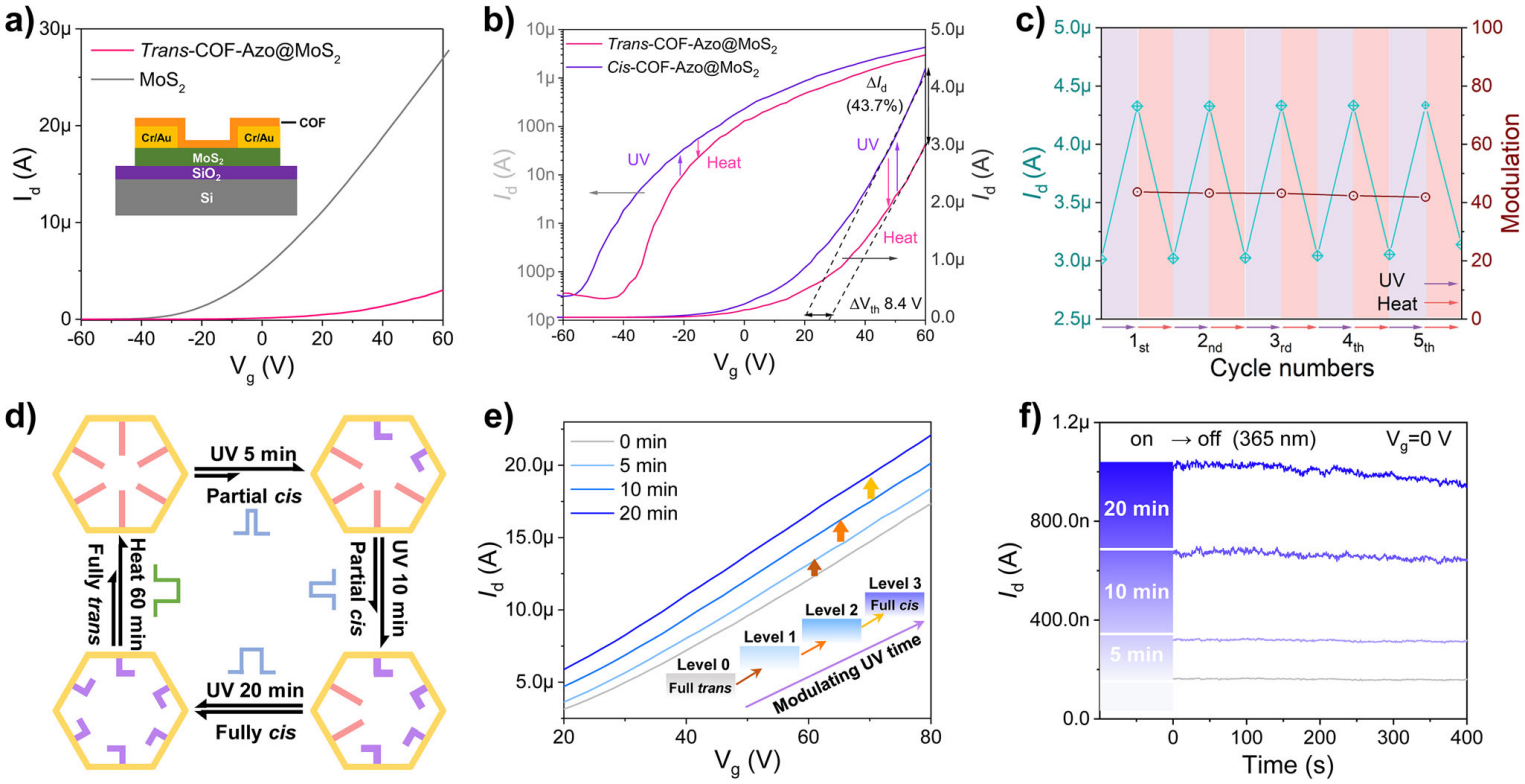
Photo‐responsive electrical behavior of COF‐Azo@MoS_2_ hybrid devices. a) Transfer characteristics of pristine MoS_2_ and *trans*‐COF‐Azo@MoS_2_ hybrid FET devices (inset: schematic illustration of the back‐gated device architecture on SiO_2_/Si with Cr/Au electrodes); b) Transfer characteristics of COF‐Azo@MoS_2_ FET device (*V*_d_ = 1 V); c) Current modulation in the hybrid COF‐Azo@MoS_2_ FET over five switching cycles. *I*_d_ was plotted at *V*_g_ = 60 V. Modulation is defined as (*I*_cis_‐*I*_trans_)/*I*_trans_ %; d) The schematic diagram of controllable partial photoisomerization to achieve multi‐level memory states; e) Stepwise transfer curves recorded after UV illumination for 5, 10, and 20 min; f) Time‐resolved channel current of the COF‐Azo@MoS_2_ FET at *V*_g_ = 0 V after UV illumination for 5, 10, and 20 min.

To investigate how the optical switching behavior of COF‐Azo films influences the electrical properties of monolayer MoS_2_, we fabricated a FET using photolithography on triangular monolayer MoS_2_ grown by chemical vapor deposition (CVD). The COF‐Azo@MoS_2_ vdWHs was then assembled through an in situ wet‐transfer process. As shown in Figure 4a, the transfer characteristics of MoS_2_ before and after the transfer of the COF‐Azo film revealed a clear modulation of the device behavior. In particular, the physisorption of COF‐Azo yielding the heterojunction is accompanied by a positive shift of the threshold voltage of about 41 V (from −35.9 to +4.9 V, determined using a constant‐current criterion of *I*_d_ = 10^−7^ A × W/L = 1.78 × 10^−7^ A) and a reduced on‐state current, indicating pronounced p‐doping in the MoS_2_ channel [37].

The UV‐light‐triggered *trans‐to‐cis* isomerization can affect the COF‐Azo electronic structure, altering the electrical characteristics of the MoS_2_ transistor. Conversely, the *cis‐to‐trans back*‐isomerization can be initiated by thermal annealing. Upon 20 min of UV irradiation, the heterojunction device exhibited a clear n‐doping modulation. As shown in Figure 4b, the drain current increased across the entire gate‐voltage range (with a 43.7% enhancement in the maximum on‐state current) and the threshold voltage shifted negatively (Δ*V*_th_ ≈ −8.4 V). Correspondingly, the channel carrier density increased by approximately 6.7 × 10^11^ cm^−2^, and the electron mobility increased from 2.43 to 2.75 cm^2^ V^−1^ s^−1^ (an increase of ∼13.2%). To further assess the reversibility and operational stability of the photoresponse, the device was subjected to repeated switching cycles consisting of 365 nm irradiation (20 min) followed by thermal annealing at 60°C (60 min). As shown in Figures 4c andS12, the COF‐Azo@MoS_2_ heterostructure maintained a stable current‐modulation amplitude of ∼41% over five consecutive cycles, confirming its robust and reversible optical‐switching behavior. The performance of the devices is comparable to or higher than many reported systems (Table S2). In contrast, the corresponding azobenzene molecular monomer (Azo‐NH_2_) failed to form a stable or well‐defined interface with MoS_2_ due to the absence of anchoring groups (Figure S13), severely hindering the reproducibility of optically switchable devices. Collectively, these results highlight the essential role of embedding azobenzene units within a rigid COF framework to achieve stable and reversible photoresponsive functionality. It should be mentioned that previous studies on photo‐switchable COFs have primarily focused on structural or optical response, and device‐level implementations remain limited (Table S3), highlighting our work that provides a device‐integrated platform to combine robust photoswitching COFs with programmable electronic functionality.

The dual response offers an effective strategy for engineering transistors with dynamically tunable threshold voltages, which is highly appealing for multi‐level memory applications. By precisely controlling the duration of UV irradiation, a stepwise increase in the *cis*‐isomer population could be achieved, which is reflected in the incremental modulation of the heterojunction's electrical output (Figure 4d), establishing multiple intermediate states between the fully *trans‐* and *cis‐* configurations. Once the electrical states of the heterojunction device are programmed by varying the UV irradiation duration time, a subsequent erase operation can be accomplished by thermal treatment, thereby enabling reversible multi‐level data storage [38]. As proof of concept (Figure 4e), 5‐ or 10‐min UV irradiation intervals were used to realize three‐level memory operation in our COF‐Azo@MoS_2_ heterojunction device. The channel current was monitored at zero gate bias under different UV pulse durations, revealing an excellent non‐volatility on the timescale of several hundreds of seconds (Figure 4f). The lifetime (t) of the memory states was extracted from the current–time decay curves using the exponential relation *I_(t)_ = I_0_ exp(‐t/τ_i_)* + *I*_∞_, where *I*_0_ and *I*_∞_ denote the initial and steady‐state currents, respectively [39]. Under zero gate bias, the current decays slowly from the on‐state to the baseline, with lifetimes across multiple levels exceeding several hundreds of seconds (Figure S14). Four additional independent devices were further fabricated, which showed consistent photoswitching behavior trends, suggesting a good reproducibility (Figure S15; Table S1). The detailed statistical data are provided in the Supporting Information. In addition, a device stored at ambient for one year retains reversible photo‐switching behavior over at least 10 consecutive cycles (Figure S16). It should be mentioned that the performance decay is primarily attributed to gradual interfacial degradation under long‐term ambient exposure, accompanied by possible azobenzene fatigue and environmental factors. In contrast, pristine MoS_2_ devices showed negligible transfer curve variation under identical conditions (Figure S17), highlighting the long‐term optical memory capability of the COF‐Azo@MoS_2_ heterojunction and its potential for multi‐level data storage and neuromorphic applications. Combined with the observed photoswitching behavior, the successful wet‐transfer of thin COF films represents a key advance over conventional in situ growth methods. By preserving the ultraflat morphology and structural integrity of COF films, this approach enables clean van der Waals interfaces, efficient interfacial coupling, and flexible integration into multi‐device architectures, thereby expanding the applicability of 2D π‐COFs in optoelectronic and memory devices.

**FIGURE 5:**
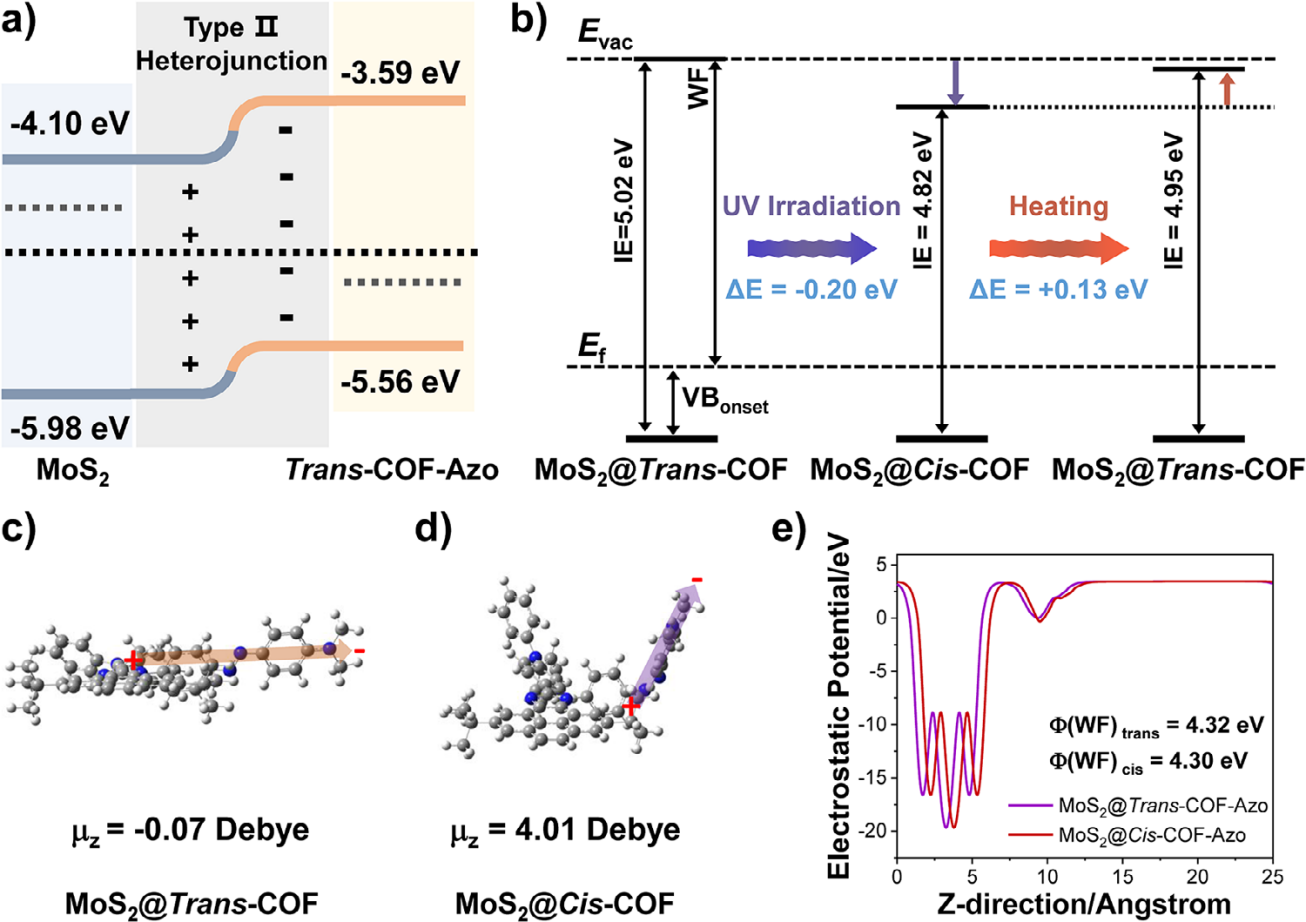
Mechanism study of photo‐responsive device. a) The formation of type II heterojunction from MoS_2_ and COF‐Azo (the shorter black slim dashed lines represent the independent Fermi level of pristine MoS_2_ and trans‐COF‐Azo before contact; the longer dotted line refer to Fermi level of MoS_2_@trans‐COF‐Azo vdWH at thermodynamic equilibrium); b) Energy‐level diagram of COF‐Azo@MoS_2_ vdWH in different isomeric states; Structural models of c) partial *trans*‐COF‐Azo fragments and d) *cis*‐COF‐Azo fragments with calculated dipole moments; e) Calculated work function of trans‐COF‐Azo@MoS_2_ and cis‐COF‐Azo@MoS_2_.

### 2.4 Mechanism of Photo‐Responsive Device

To further elucidate the interfacial electronic modulation mechanism, x‐ray photoelectron spectroscopy (XPS) analyses were performed. As shown in Figure S18, XPS spectra showed that the electron depletion (*p*‐doping) of MoS_2_ resulted in a shift of 0.1 eV for the Mo 3d peaks toward lower binding energy. The p‐type doping behavior of the COF‐Azo@MoS_2_ vdWH device arises from the interfacial doping induced by charge transfer during the formation of a type‐II staggered heterojunction, where the Fermi Level of MoS_2_ is substantially higher than that of *trans*‐COF‐Azo (Figure 5a). Upon contact, electrons flow from MoS_2_ to *trans*‐COF‐Azo, partially depleting electron carriers in the MoS_2_ channel until Fermi‐level equilibrium is established.

Ultraviolet photoelectron spectroscopy (UPS) measurements were conducted for the COF‐Azo@MoS_2_ vdWH device to study the observed n‐doping response resulted from the *trans‐ to‐ cis* photoisomerization. As shown in Figure S19, the UPS results indicate that the secondary electron cutoff (SECO) shifts of −0.20 eV upon illumination and +0.13 eV upon heating, corresponding to a decrease and increase in work function (WF), while the valence‐band onset remained unchanged. Figure 5b summarized corresponding energy‐level diagram of COF‐Azo@MoS_2_ vdWH upon the illumination and heating treatment, suggesting the molecular dipole moment and orientation changes during isomerization, which induced a work function tuning and thus impacted the optoelectronic performance.

Density functional theory (DFT) calculations reveal a pronounced dipole reorientation associated with the photoisomerization of COF‐Azo. As illustrated in Figure 5c, in the *trans* configuration, the Azo moiety tethered to the COF side chain adopts a nearly planar geometry parallel to the MoS_2_ surface, giving rise to a negligible out‐of‐plane dipole component. Upon illumination, COF‐Azo undergoes *trans‐cis* isomerization (Figure 5d), which lifts the dimethylamine‐terminated phenyl ring away from the substrate and increases the vertical dipole moment to 4.01 D. This upward‐oriented dipole induces electronic doping of MoS_2_, resulting in the enhanced electronic performance in heterojunction. Figures 5e and S20 present the calculated change in work function induced by the isomerization of a simplified COF fragment. Following the *trans‐cis* conversion, the work function decreases by approximately ∼0.02 eV. This modest shift likely arises from use of an isolated segment, which underestimates the collective dipole contribution present in the extended COF‐Azo framework. Nevertheless, the calculated trend aligns well with the direction of the experimentally observed WF modulation, supporting a dipole‐driven mechanism for interfacial electronic regulation in the COF‐Azo@MoS_2_ heterostructure.

## 3 Conclusion

In summary, a “wet transfer of in situ grown COF films” strategy was developed to fabricate an azobenzene‐containing COF‐Azo film, which was further integrated with monolayer MoS_2_ to construct a robust photo‐responsive hybrid heterostructure device. We showed that the COF‐Azo layer integrated in COF‐Azo@MoS_2_ hybrid transistors undergoes a reversible *trans‐cis* photoisomerization, enabling controllable modulation of drain current, threshold voltage, and carrier density under alternating UV irradiation and thermal annealing. The hybrid device exhibits robust and reproducible switching behavior over multiple cycles, confirming its stable photoelectronic response. Photoemission analyses revealed reversible shifts in work function induced by photoisomerization and thermal recovery, consistent with dipole modulation. These findings establish a structurally designable and highly robust COF framework that overcomes the limited stability of small‐molecule photochromic systems when integrated into working devices. By enabling facile integration, large‐area fabrication, and efficient three‐dimensional charge transport, this architecture opens a new route for building photo‐switchable 2D semiconductor interfaces and advancing next‐generation optoelectronic devices. Future efforts will focus on systematically addressing the key practical metrics for COF‐based non‐volatile memory devices, including long‐term operational stability, endurance, and scalability.

## Author Contributions

**Kexin Wang**: investigation, writing – original draft, writing – review and editing. **Bin Han**: investigation, writing – review and editing, writing – original draft. **Fan Qiu**: investigation. **Yeonsu Jeong**: investigation. **Yusheng Chen**: investigation. **Yubin Fu**: investigation, supervision. **Christos Gatsios**: investigation. **Marco Vittorio Nardi**: investigation, supervision. **Melanie Timpel**: investigation, supervision. **Yang Hou**: writing – original draft, writing – review and editing, supervision. **Shun‐Qi Xu**: conceptualization, methodology, writing – review and editing, writing – original draft, supervision. **Paolo Samorì**: conceptualization, methodology, writing – original draft, writing – review and editing, supervision.

## Conflicts of Interest

The authors declare no conflicts of interest.

## Supporting information



The authors have cited additional references within the Supporting Information. **Supporting File**: anie72566‐sup‐0001‐SuppMat.docx.

## Data Availability

The data that support the findings of this study are available from the corresponding author upon reasonable request.
