# Supplementary material for: Wet Transfer of In Situ Grown Azo‐Containing Two‐Dimensional Conjugated Covalent Organic Framework Films for Photoswitchable Electronic Devices
Source: Angew Chem Int Ed Engl. 2026 Jun 7;65(32):e2260268. doi: 10.1002/anie.2260268 (PMC13427172; doi:10.1002/anie.2260268)
Supplement: Supplementary file 1 — The authors have cited additional references within the Supporting Information. Supporting File: anie72566‐sup‐0001‐SuppMat.docx. [file ANIE-65-e2260268-s001.docx]

Wet Transfer of In-situ Grown Azo-Contained Two-dimensional Conjugated Covalent Organic Frameworks Films for Photo-switchable Electronic Devices

Kexin Wang,^a,c^ Bin Han,^a^ Fan Qiu,^b^ Yeonsu Jeong,^a^ Yusheng Chen ^a^ Yubin Fu,^b^ Christos Gatsios ^d^ Marco Vittorio Nardi,^d^ Melanie Timpel,^d^ Yang Hou,*^,c^ Shun-Qi Xu,*^,a,b^ and Paolo Samorì*^,a^

Kexin Wang, Bin Han, Yusheng Chen, Yeonsu Jeong, Shun-Qi Xu, Paolo Samorì

University of Strasbourg & CNRS, ISIS & icFRC

8 Allée Gaspard Monge, Strasbourg F-67000, France
E-mail: [samori@unistra.fr](mailto:samori@unistra.fr)

Fan Qiu, Yubin Fu, Shun-Qi Xu
School of Energy and Environment, Southeast University

Nanjing 211189, China

E-mail: [shunqi.xu@seu.edu.cn](mailto:shunqi.xu@seu.edu.cn)

Kexin Wang, Yang Hou
Key Laboratory of Biomass Chemical Engineering of Ministry of Education

College of Chemical and Biological Engineering

Zhejiang University, Hangzhou 310 027, China

E-mail: [yhou@zju.edu.cn](mailto:yhou@zju.edu.cn)

Christos Gatsios, Marco Vittorio Nardi, Melanie Timpel

IMEM-CNR Institute of Materials for Electronics and Magnetism, Trento Unit c/o Fondazione Bruno Kessler, Via alla Cascata 56/C, 38123 Trento, Italy

Equal contribution: Kexin Wang and Bin Han equally contributed to this work.

.

**Experimental Section**

**Materials**

All reagents were obtained from commercial sources and used as received. 4,4',4''-Nitrilotribenzaldehyde (N-TBA), 2,7-Di-tert-butylpyrene-4,5,9,10-tetraone (t-BuPy-tetraone), *N,N*-Dimethyl-4,4-azodianiline (Azo-NH_2_), and benzyl alcohol (BA) were purchased from BLDpharm. Ammonium acetate (CH_3_COONH_4_), acetic acid (HOAc), tetrahydrofuran (THF), ethanol, and *N,N*-dimethylformamide (DMF) were acquired from Sigma-Aldrich. Mesitylene (Mes) was obtained from Thermal Fisher. Chemical vapor deposited MoS_2_ flakes were purchased from Six Carbon Technology. Si/SiO_2_ n^++^ substrates with a 270 nm thick oxide layer were purchased from Fraunhofer Institute for Photonic Microsystems (IPMS). The 2 mm thick quartz substrates were purchased from Plan Optik.

**Fabrication and Electrical Characterization of COF-Azo@MoS_2_ Hybrid FETs**

Monolayer MoS₂ flakes grown by chemical vapor deposition were transferred onto SiO_2_/Si substrates. Back-gated field-effect transistors (FETs) were defined by laser writer lithography. Source and drain electrodes composed of chromium (10 nm) and gold (60 nm) were thermally evaporated, followed by lift-off in warm acetone. The COF-Azo film was subsequently transferred onto the fabricated MoS_2_ FETs, rinsed with warm acetone, and annealed under vacuum at 100 °C overnight to remove residual adsorbates before measurement.

Electrical measurements were conducted in an N₂-filled glove box using a probe station connected to a Keithley 2636 source-measure unit. All measurements were performed in the dark to eliminate photoconductive effects of MoS_2_.

Photo-switching of COF-Azo was induced using a fiber-coupled light-emitting diode (LED) with a wavelength of λ = 365 nm, 1.2 mW cm^-2^ for 20 min, while thermal switching was achieved by heating the device to 60 °C for 60 min. All the data were collected after operating the devices two or three times.

The field-effect mobility (μ) was extracted from the linear regime of the transfer characteristics using:

$$\mu=\frac{{dI}_{d}}{dV_{g}} \times\frac{L}{WC_{i}V_{d}}$$

where L and W are the channel length and width, respectively, C_i_ is the capacitance per unit area (12.79 nF/cm^2^ for SiO_2_), I_d_ is the drain current, and V_g_ and V_d_ are the gate and drain voltages, respectively.

The change in charge carrier density upon doping was calculated as:

$$\Delta n=\frac{C_{i}{\Delta V}_{th}}{e}$$

where Δ*V*_th_ is the threshold-voltage shift before and after doping and e=1.602×10^-19^ C is the elementary charge.

**Characterization Techniques**

**X-ray diffraction (XRD).**

Powder XRD patterns were collected on a Bruker D8 diffractometer using Cu Kα radiation (λ = 1.5406 Å; 40 kV, 40 mA).

**Transmission electron microscopy (TEM).**

High-resolution TEM images were obtained using a Thermo Fisher Scientific Talos F200X G2 microscope operated at 200 kV.

**Fourier-transform infrared spectroscopy (FT-IR).**

FT-IR spectra were recorded on a Bruker INVENIO R spectrometer equipped with a liquid-nitrogen-cooled MCT detector.

**Solid-state nuclear magnetic resonance (ssNMR).**

The ^13^C CP/MAS NMR spectra of the COFs were recorded on an Agilent DD2 600 Solid NMR System with 3.2 mm zirconia rotors. The spinning rate is 8 kHz and the contact time is 3 ms.

**Atomic force microscopy (AFM).**

AFM imaging was carried out on a Bruker Dimension Icon microscope operated in tapping mode under ambient conditions using TESPA-V2 tips (spring constant ≈ 42 N m⁻¹).

**UV–visible absorption spectroscopy.**

UV–vis absorption spectra were recorded at room temperature on a JASCO V-650 spectrophotometer. Photoswitching experiments were conducted with optical fiber-coupled LEDs (Thorlabs; λ = 365 nm, 1.2 mW cm^-2^). Thermal switching was achieved using a heating plate at ca. 60 °C.

**Raman spectroscopy.**

Raman spectra were recorded in air using a Renishaw inVia Raman spectrometer equipped with a 532 nm laser. The laser power was maintained below 1 mW to avoid local heating. The spectral resolution was 1 cm^-1^.

**X-ray photoelectron spectroscopy (XPS).**

XPS measurements were carried out on a Thermo Scientific K-Alpha spectrometer with an Al Kα source (1486.6 eV). The base pressure during measurement was ~10⁻⁹ mbar. Binding energies were calibrated to the C 1s peak at 284.8 eV. Data fitting and chemical assignment were performed using Avantage software.

**Ultraviolet photoelectron spectroscopy (UPS).**

UPS measurements were conducted under ultra-high vacuum (≈4.5 × 10^-9^ mbar). All the data were collected for the *trans-* and *cis-* configurations of the MoS_2_@COF-Azo by illuminating the sample with a light-emitting diode (LED) with a wavelength of λ = 365 nm (1.2 mW cm^-2^) for 20 min, while thermal switching was achieved by heating the device to 60 ^o^C for 60 min. The ionization energy (IE) was calculated from the secondary-electron cutoff (SECO) and valence-band onset (VB_onset_) as:

IE = WF + VB_onset_

where WF is the sample work function determined from the SECO.

**Thermogravimetric and differential scanning calorimetry (TGA / DSC).**

Thermal analyses were carried out on a Mettler Toledo TGA/DSC 2 instrument under a nitrogen atmosphere, with a heating rate of 10 °C min^-1^ from 50 °C to 800 °C.

**Water contact angle.**

Static water contact angles were measured with a Krüss DSA 100 goniometer by depositing a 2 μL droplet of Milli-Q water onto the UV/heat-treated COF film surface.

**Cyclic voltammetry (CV).**

CV was performed in a three-electrode configuration using an electrochemical workstation (PGSTAT204). The working electrode was a COF-Azo film transferred onto an Au (20 nm)-coated Si/SiO₂ substrate, with a Pt wire and an Ag/AgCl electrode serving as the counter and reference electrodes, respectively. The electrolyte was 0.1 M tetrabutylammonium hexafluorophosphate (TBAPF_6_) in anhydrous acetonitrile, which was degassed by bubbling N₂ for 20 min prior to measurement. The scan rate was fixed at 100 mV s^-1^. The ferrocene/ferrocenium (Fc/Fc⁺) redox couple was used as an internal reference.

To obtain the *trans* and *cis* states, CV measurements were first performed on the pristine (*trans*) COF-Azo film. The same film was then rinsed with acetonitrile and acetone, irradiated with UV light (365 nm, 1.2 mW cm^-2^) for 20 min to induce cis isomerization, and subsequently measured again under identical electrochemical conditions. The HOMO energy level (E_HOMO_) was estimated from the onset of oxidation as:

E_HOMO_ (eV) = - (E_ox, onset_ - E_Fc/Fc+_) - 4.80 eV

Where E_ox, onset_ and E_Fc/Fc+_ are measured vs. the same reference electrode.

**Author contributions**

P. S. and S.X. conceived the idea. S.X. designed the structure of COFs. K.W. performed most experiments and analyzed the data under the guidance of S.X., B.H. conducted the XPS, AFM, and contact angle analyses. KX performed electrical test under the guidance of B H & S. X., F.Q. carried out the solid-state NMR measurements. Y.F. performed the DFT calculations. C.G., M.V.N., and M.T. conducted the UPS measurements and data analysis. C.Y. and Y.J. provided valuable suggestions. K.W., S.X., Y.H., B.H., and P.S. co-wrote the manuscript. All authors discussed the results and commented on the manuscript.

**Synthesis of COF-Azo powder and film**

A mixture of 4,4′,4″-nitrilotribenzaldehyde (10.5 mg, 0.031 mmol), 2,7-di-tert-butylpyrene-4,5,9,10-tetraone (17.9 mg, 0.047 mmol), N,N-dimethyl-4,4′-azodianiline (34.5 mg, 0.144 mmol), and ammonium acetate (7.4 mg, 0.096 mmol) was weighted into a glass tube. Benzyl alcohol (0.8 mL), mesitylene (0.2 mL), and acetic acid (6 M, 0.1 mL) were added as the solvent mixture. The tube was flash-frozen in liquid nitrogen, evacuated under reduced pressure, and the freeze–pump–thaw cycle was repeated three times; the tube was then sealed after evacuation. Upon warming to room temperature, the sealed tube was heated at 120 °C for 3 days. The resulting orange precipitate was collected by centrifugation, washed successively with DMF, THF, and acetone over several days, and dried under vacuum at 100 °C for 12 h to yield COF-Azo as an orange powder.

**Scheme 1**. Synthesis of COF-Azo.

Following the same procedure, COF-Azo films were directly grown on smooth, face-down SiO_2_ substrates. After reaction, the films were washed thoroughly with DMF, THF, and acetone for several days and dried under a nitrogen stream. A thin polystyrene layer was spin-coated on the COF surface, and the composite was floated on distilled water to detach the SiO_2_ substrate. The freestanding COF-Azo films were subsequently transferred onto quartz plates or FET devices for photoisomerization and electrical characterization.

**Figure S1**. XRD patterns of COA-Azo and its corresponding monomers.

**Figure S2**. Structural model of COF-Azo with AA stacking.

**Figure S3**. Structural model of COF-Azo with AB stacking.

**Figure S4**. Structural model of COF-Azo with inclined AA stacking.

**Figure S5**. HRTEM image of COF-Azo.

**Figure S6.** (a) CO_2_ adsorption-desorption isotherms for COF-Azo at 273 K; (b) The pore size distribution calculated from the isotherm linear plot.

**Figure S7.** TGA and DSC curves of COF-Azo and the azobenzene monomer.

**Figure S8.** Step height measurement of the upper surface of COF-Azo film. This upper surface is a non-contact surface, used here for comparison.

**Figure S9.** FT-IR spectra of COF-Azo and its corresponding film.

**Figure S10**. UV-Vis absorption spectra of the COF-Azo film after (a) UV illumination or (b) thermal treatment for different durations.

**Figure S11**. Cyclic voltammograms of (a) *trans*-COF-Azo film, (b) *cis*-COF-Azo film, and (c) ferrocene (Fc). (d) Optical band gaps of the trans- and cis-COF-Azo films determined from UV-Vis absorption spectra.

**Figure S12.** Transfer curves of the hybrid COF-Azo@MoS_2_ FET for five switching cycles (*trans*-*cis* transition).

**Figure S13.** (a) Optical microscope image of a MoS_2_ device coated with drop-cast Azo-NH_2_ molecules (0.2 mg/mL in ethanol). (b) Transfer Curves of the MoS_2_@Azo-NH_2_ device measured after UV irradiation and subsequent heating.

**Figure S14.** Linear decay curve and fitting line of channel current of the COF-Azo@MoS_2_ FET after UV illumination for 0 (a), 5 (b), 10 (c), and 20 min (d).

**Figure S15.** The Photo-switch behavior of another four independent devices.

**Table S1.** Statistical data analysis for another 4 independent devices

| Device number | Mobility  (cm^2^ V^-1^ s^-1^) | | Mobility Change  Percentage (%) | Modulation amplitude  (%) | Threshold voltage shift  (V) |
| --- | --- | --- | --- | --- | --- |
| 1 | trans | ~13.2 | 17.4 | 40.5 | ~17.2 |
|  | cis | ~15.5 |  |  |  |
| 2 | trans | ~8.9 | 16.8 | 38.5 | ~16.1 |
|  | cis | ~10.4 |  |  |  |
| 3 | trans | ~17.5 | 10.3 | 30.8 | ~13.9 |
|  | cis | ~19.3 |  |  |  |
| 4 | trans | ~8.6 | 10.5 | 38.2 | ~15.3 |
|  | cis | ~9.5 |  |  |  |
| Statical  Average | N/A | N/A | 13.7±3.8 | 37±4.3 | 15.6±1.4 |

The average mobility change is (13.7 ± 3.8)%, the modulation amplitude is (37 ± 4.3)%, and the threshold voltage shift is (15.6 ± 1.4) V. These results indicate a reasonable device-to-device variation. The observed variations can be attributed to several factors: (1) Differences in the effective MoS_2_ channel area may introduce variability in device performance (this can be further minimized in future studies by defining the channel geometry through lithographic or etching processes); (2) Slight variations in the thickness of the transferred COF films may influence interfacial coupling; (3) Although the COF/MoS_2_ interface is atomically flat, the upper surface of the COF film exhibits some roughness, and non-uniform particle distribution may lead to local variations in charge transport.

**Figure S16**. The cycling stability and reversibility of a COF-Azo@MoS_2_ FET device stored at ambient conditions for one year. (a) Transfer characteristics of COF-Azo@MoS_2_ FET device over ten switching cycles; (b) Ten switching cycles of current modulation in the hybrid COF-Azo@MoS_2_ FET; (c) Stepwise transfer curves recorded after UV illumination for 5, 10, 15, and 20 min, followed by thermal treatment; (d) Time-resolved channel current of the COF-Azo@MoS_2_ FET at Vg = 0 V after UV illumination for 5, 10, 15, and 20 min.

**Figure S17.** (a) Stepwise transfer curves recorded after UV illumination for 5, 10, and 20 min. (b). Time-resolved channel current of the MoS_2_ FET at Vg = 0 V after UV illumination for 5, 10, and 20 min.

**Figure S18.** XPS spectra of Mo 3d of pristine MoS_2_ and COF-Azo@MoS_2_.

**Figure S19.** UPS spectra of COF-Azo@MoS_2_ after illumination and subsequent thermal treatment during switching cycles, showing (a) the secondary electron cutoff (SECO) and (b) the valence band (VB) region.

**Figure S20.**  Structural models of partial *trans*-COF-Azo fragments (left) and *cis*-COF-Azo fragments (right) on MoS_2_.

**Table S2**. A brief summary of recently reported photo-switchable FET devices.

| System | Photoswitchable species | Preparation  method | Mobility Change  Percentage (%) | Modulation (%) @ V_G_ (V), V_D_ (V) | ΔVth (V) | Memory Properties | Literature. |
| --- | --- | --- | --- | --- | --- | --- | --- |
| **COF-Azo@MoS_2_** | **COF-Azo film** | **Wet-transfer** | **13.2** | **41 @ 60, 1** | **8.4** | **Multi-level**  **Non-volatile** | **This work** |
| POMPYA 1 | Azo and aryl-Azo | Spin-coating | 40 (state 0-1) | 39.5 (state0-1) @ -60, -60 | N/A | Three states | *Adv. Mater.* **2021,** *33*, 2005613 |
| Azobenzene+  MoS_2_ | Azo | Drop-cast /  microfluidic | N/A | N/A | 11 ^a^ | Molecular switch on TMD channel | *Nanotechnology*. **2024**, *35*, 395501. |
| MoS_2_/ CoPc(TFAP) | Azo coordiinated CoPc | Immersion and self-assembly | N/A | 25.9 @ 40, 1 ^a^ | 5.6 ^a^ | Programmable logic‑memory | *ACS Nano* **2021**, *15*, 13732. |
| Azo-BTBT- | Azo-BTBT-8 | Spin-coating | 8.4 times | 10 times @  -25, -25 | 0.48 ^a^ | N/A | *Nat. Commun.*  **2022***, 13,* 4912. |
| Azo-CNM/GFETGr | Azo-CNM | transfer | N/A | N/A | N/A | N/A | *Chem. Eur. J.* **2020***, 26,* 6473. |
| Functioanlized azobenzene graphene | Azobenzene via diazonium chemistry | In situ covalent functionalization | N/A | 46 @ 12, N/A  (conductivity) | N/A | N/A | *Adv. Electron. Mater*. **2018**, *4*, 1800021 |
| Azo/MoS_2_ | Azo molecules | Spin-coating | 22 | 34 @ 60, 0.1 | 18.48 | Multibit nonvolatile | *ACS Nano* **2019**, *13*, 4814. |
| WSe_2_/DAE | DAE | Spin-coating | 60.8 | 61 @ 30, 2 | 2.5 | Multilevel nonvolatile | *Adv. Mater.* **2019**, *31*, 1903402. |
| MoS_2_-GNR VDWH | SP-C18 | Spin-coating | N/A | 52 @ 30, 1 | 2.2 ^a^ | Multilevel nonvolatile | *Adv. Mater.* **2020**, *32*, 2001268 |

^a^: estimated from the figures in the related literatures

**Table S3**. A brief summary of the photoswitchable COFs

| Types of COFs | Powder or Films | Switching Cycles | Literature |
| --- | --- | --- | --- |
| Azobenzene-contained COFs | powder | 4 | *Adv. Funct. Mater.* **2023**, *33*, 2302225 |
|  | powder | 3 | *Angew. Chem. Int. Ed.* **2024**, *63*, e202400009 |
|  | film | 3 | *Sci. Adv.* **2025**, *11*, eadw8452 |
| Diarylethene-contained COFs | powder | 2 | *Chem. Mater*. **2022**, *34*, 1956-1964 |
|  | powder | - | *Nat. Commun,* **2024**, *15*, 1479 |
|  | film | 2.5 | *Angew. Chem. Int. Ed*. **2019**, *58*, 16101-16104 |
| Spiropyran-contained COFs | powder | 5 | *Nat. Commun*, **2023**, *14*, 3765 |

**Supplementary Table 1.** Fractional atomic coordinates for the incline (AA) stacking

Crystal system: Hexagonal

Space group: P_6_

a= 35.3 Å, b= 35.3 Å, c= 5.8 Å

α= 90^o^, β = 90^o^, γ = 120^o^

**Table S4. Cartesian coordinates for the optimized AA stacking model**

| C1 | C | 2.6424 | -1.64622 | -0.04345 |
| --- | --- | --- | --- | --- |
| C2 | C | 2.64927 | -1.6149 | -0.21489 |
| C3 | C | 2.62449 | -1.59375 | -0.2168 |
| C4 | C | 2.59215 | -1.60335 | -0.04713 |
| C5 | C | 2.58508 | -1.63471 | 0.12404 |
| C6 | C | 2.60986 | -1.65592 | 0.12549 |
| C7 | C | 2.56805 | -1.5813 | -0.04839 |
| C8 | C | 2.58816 | -1.53603 | -0.05098 |
| C9 | C | 2.55548 | -1.52509 | -0.04799 |
| C10 | C | 2.51515 | -1.56417 | -0.0491 |
| N11 | N | 2.52391 | -1.59798 | -0.04461 |
| C12 | C | 2.55955 | -1.48321 | -0.04253 |
| C13 | C | 2.52084 | -1.48027 | -0.04761 |
| C14 | C | 2.52416 | -1.43809 | -0.05165 |
| C15 | C | 2.60073 | -1.44467 | -0.02542 |
| C16 | C | 2.60544 | -1.40249 | -0.01891 |
| C17 | C | 2.56663 | -1.40067 | -0.05491 |
| C18 | C | 2.64905 | -1.36108 | 0.03068 |
| C19 | C | 2.66732 | -1.33347 | -0.19359 |
| C20 | C | 2.6854 | -1.37002 | 0.12918 |
| C21 | C | 2.64226 | -1.33265 | 0.21606 |
| C22 | C | 2.49519 | -1.64183 | -0.03952 |
| C23 | C | 2.46903 | -1.66189 | 0.15669 |
| C24 | C | 2.43825 | -1.70699 | 0.15646 |
| C25 | C | 2.43338 | -1.733 | -0.03888 |
| C26 | C | 2.46141 | -1.71349 | -0.22882 |
| C27 | C | 2.49183 | -1.66835 | -0.22975 |
| N28 | N | 2.40228 | -1.77566 | -0.05716 |
| N29 | N | 2.37134 | -1.79763 | 0.09101 |
| C30 | C | 2.33903 | -1.83973 | 0.06889 |
| C31 | C | 2.30788 | -1.85767 | 0.2475 |
| C32 | C | 2.27305 | -1.9011 | 0.2341 |
| C33 | C | 2.26853 | -1.92772 | 0.04204 |
| C34 | C | 2.2997 | -1.9095 | -0.13754 |
| C35 | C | 2.33463 | -1.86609 | -0.12421 |
| N36 | N | 2.23501 | -1.97005 | 0.03129 |
| C37 | C | 2.22878 | -2.00104 | 0.21022 |
| C38 | C | 2.20469 | -1.98524 | -0.15903 |
| H39 | H | 2.67232 | -1.60746 | -0.34042 |
| H40 | H | 2.6302 | -1.57136 | -0.34392 |
| H41 | H | 2.56223 | -1.64205 | 0.25049 |
| H42 | H | 2.60427 | -1.67834 | 0.25198 |
| H43 | H | 2.6212 | -1.51496 | -0.05271 |
| H44 | H | 2.62792 | -1.44758 | -0.01538 |
| H45 | H | 2.57008 | -1.37077 | -0.08084 |
| H46 | H | 2.67276 | -1.35235 | -0.32533 |
| H47 | H | 2.69842 | -1.30359 | -0.16101 |
| H48 | H | 2.64457 | -1.32372 | -0.26096 |
| H49 | H | 2.69578 | -1.3856 | 0.00065 |
| H50 | H | 2.67341 | -1.39119 | 0.28073 |
| H51 | H | 2.71459 | -1.33973 | 0.1809 |
| H52 | H | 2.62256 | -1.3532 | 0.35881 |
| H53 | H | 2.6255 | -1.31657 | 0.14358 |
| H54 | H | 2.67326 | -1.30665 | 0.28459 |
| H55 | H | 2.4717 | -1.64347 | 0.29837 |
| H56 | H | 2.41889 | -1.72053 | 0.29819 |
| H57 | H | 2.45949 | -1.73196 | -0.36905 |
| H58 | H | 2.5115 | -1.65477 | -0.37047 |
| H59 | H | 2.31024 | -1.83915 | 0.38845 |
| H60 | H | 2.25076 | -1.91319 | 0.36538 |
| H61 | H | 2.29734 | -1.92793 | -0.27867 |
| H62 | H | 2.35675 | -1.85391 | -0.25633 |
| H63 | H | 2.22937 | -2.02922 | 0.13632 |
| H64 | H | 2.19713 | -2.01248 | 0.29351 |
| H65 | H | 2.25412 | -1.98718 | 0.34302 |
| H66 | H | 2.20705 | -2.01087 | -0.25227 |
| H67 | H | 2.21067 | -1.95921 | -0.28087 |
| H68 | H | 2.17125 | -1.99875 | -0.09536 |
| N69 | N | 0.33333 | 0.66667 | -0.04218 |

**Table S5. Cartesian coordinates for the optimized AB stacking model**

Crystal system: Hexagonal

Space group: P63

a= 35.1 Å, b= 35.1 Å, c= 11.2 Å

α= 90^o^, β = 90^o^, γ = 120^o^

| C1 | C | 0.97964 | 1.02481 | 0.27725 |
| --- | --- | --- | --- | --- |
| C2 | C | 0.99288 | 1.06042 | 0.19692 |
| C3 | C | 0.97132 | 1.08509 | 0.19376 |
| C4 | C | 0.93565 | 1.07507 | 0.27111 |
| C5 | C | 0.92306 | 1.04032 | 0.35364 |
| C6 | C | 0.94448 | 1.0155 | 0.35606 |
| C7 | C | 0.9133 | 1.09919 | 0.26443 |
| C8 | C | 0.93445 | 1.14487 | 0.26334 |
| C9 | C | 0.90264 | 1.15721 | 0.25254 |
| C10 | C | 0.86176 | 1.11851 | 0.24631 |
| N11 | N | 0.86931 | 1.08388 | 0.25564 |
| C12 | C | 0.90753 | 1.19968 | 0.24644 |
| C13 | C | 0.8693 | 1.20338 | 0.2319 |
| C14 | C | 0.87324 | 1.24592 | 0.22199 |
| C15 | C | 0.94901 | 1.23825 | 0.2541 |
| C16 | C | 0.95452 | 1.28088 | 0.24266 |
| C17 | C | 0.91592 | 1.28305 | 0.22028 |
| C18 | C | 0.9992 | 1.3227 | 0.25025 |
| C19 | C | 1.01391 | 1.34077 | 0.12145 |
| C20 | C | 1.03647 | 1.31584 | 0.30581 |
| C21 | C | 0.99663 | 1.35878 | 0.327 |
| C22 | C | 0.83884 | 1.04038 | 0.2525 |
| C23 | C | 0.81348 | 1.01888 | 0.35465 |
| C24 | C | 0.78 | 0.97453 | 0.34833 |
| C25 | C | 0.77146 | 0.95095 | 0.23986 |
| C26 | C | 0.79805 | 0.97198 | 0.13949 |
| C27 | C | 0.83147 | 1.01632 | 0.14559 |
| N28 | N | 0.73811 | 0.90926 | 0.22541 |
| N29 | N | 0.70833 | 0.88587 | 0.30409 |
| C30 | C | 0.67393 | 0.84495 | 0.28609 |
| C31 | C | 0.64303 | 0.82558 | 0.37914 |
| C32 | C | 0.6053 | 0.78404 | 0.36382 |
| C33 | C | 0.59773 | 0.76051 | 0.2555 |
| C34 | C | 0.62942 | 0.7797 | 0.16344 |
| C35 | C | 0.66696 | 0.8214 | 0.17822 |
| N36 | N | 0.56076 | 0.7204 | 0.23989 |
| C37 | C | 0.5481 | 1.68547 | 0.32588 |
| C38 | C | 0.53287 | 0.71152 | 0.13669 |
| H39 | H | 1.0183 | 1.06843 | 0.13893 |
| H40 | H | 0.98167 | 1.1102 | 0.13321 |
| H41 | H | 0.89829 | 1.03279 | 0.41343 |
| H42 | H | 0.93423 | 0.99037 | 0.41636 |
| H43 | H | 0.96767 | 1.16544 | 0.26924 |
| H44 | H | 0.97583 | 1.23489 | 0.26706 |
| H45 | H | 0.91935 | 1.31278 | 0.20125 |
| H46 | H | 1.01511 | 1.3159 | 0.06541 |
| H47 | H | 1.04652 | 1.37019 | 0.12224 |
| H48 | H | 0.9912 | 1.34961 | 0.08071 |
| H49 | H | 1.04422 | 1.29542 | 0.24858 |
| H50 | H | 1.02658 | 1.30017 | 0.39338 |
| H51 | H | 1.06697 | 1.34706 | 0.31694 |
| H52 | H | 0.98131 | 1.34519 | 0.4132 |
| H53 | H | 0.97758 | 1.3715 | 0.28307 |
| H54 | H | 1.0292 | 1.38721 | 0.34216 |
| H55 | H | 0.81891 | 1.03566 | 0.43334 |
| H56 | H | 0.76163 | 0.9598 | 0.42288 |
| H57 | H | 0.79288 | 0.95531 | 0.06063 |
| H58 | H | 0.85001 | 1.03118 | 0.07134 |
| H59 | H | 0.64752 | 0.84189 | 0.45825 |
| H60 | H | 0.58307 | 0.77117 | 0.43213 |
| H61 | H | 0.62534 | 0.76331 | 0.08464 |
| H62 | H | 0.689 | 0.83446 | 0.10949 |
| H63 | H | 0.54462 | 1.65613 | 0.28185 |
| H64 | H | 0.51666 | 1.67737 | 0.3665 |
| H65 | H | 0.57213 | 0.69404 | 0.39757 |
| H66 | H | 0.53355 | 1.6862 | 0.08158 |
| H67 | H | 0.54282 | 0.74055 | 0.08088 |
| H68 | H | 0.49902 | 0.69985 | 0.16487 |
| C69 | C | 0.69716 | 1.32004 | 0.17261 |
| C70 | C | 0.69609 | 1.29101 | 0.08358 |
| C71 | C | 0.72562 | 1.27512 | 0.08733 |
| C72 | C | 0.7567 | 1.28751 | 0.18088 |
| C73 | C | 0.75937 | 1.31827 | 0.26654 |
| C74 | C | 0.7299 | 1.33439 | 0.26225 |
| C75 | C | 0.78203 | 1.26674 | 0.19145 |
| C76 | C | 0.76171 | 1.22136 | 0.19109 |
| C77 | C | 0.79397 | 1.2102 | 0.20585 |
| C78 | C | 0.83444 | 1.24916 | 0.21231 |
| N79 | N | 0.82605 | 1.28342 | 0.20704 |
| C80 | C | 0.78915 | 1.16803 | 0.2133 |
| C81 | C | 0.82727 | 1.16419 | 0.22593 |
| C82 | C | 0.82293 | 1.12147 | 0.23139 |
| C83 | C | 0.74737 | 1.13005 | 0.20962 |
| C84 | C | 0.74134 | 1.08731 | 0.21339 |
| C85 | C | 0.78007 | 1.08438 | 0.22097 |
| C86 | C | 0.69473 | 1.04815 | 0.21326 |
| C87 | C | 0.66835 | 1.05009 | 0.10306 |
| C88 | C | 0.67135 | 1.05038 | 0.32973 |
| C89 | C | 0.69213 | 1.00221 | 0.20967 |
| C90 | C | 0.85449 | 1.32747 | 0.21583 |
| C91 | C | 0.87709 | 1.34651 | 0.32355 |
| C92 | C | 0.90592 | 1.39224 | 0.33193 |
| C93 | C | 0.91251 | 1.41987 | 0.23286 |
| C94 | C | 0.88865 | 1.40109 | 0.12713 |
| C95 | C | 0.86032 | 1.3554 | 0.11819 |
| N96 | N | 0.94155 | 1.46356 | 0.23179 |
| N97 | N | 0.96325 | 1.48793 | 0.3222 |
| C98 | C | 0.99449 | 1.53096 | 0.31617 |
| C99 | C | 1.00953 | 1.55455 | 0.42406 |
| C100 | C | 1.04295 | 1.599 | 0.4236 |
| C101 | C | 1.06309 | 1.62096 | 0.31592 |
| C102 | C | 1.04714 | 1.5973 | 0.20778 |
| C103 | C | 1.01326 | 1.55302 | 0.20762 |
| N104 | N | 1.09704 | 1.66358 | 0.31633 |
| C105 | C | 1.09424 | 0.69779 | 0.3803 |
| C106 | C | 1.13748 | 1.67612 | 0.25303 |
| H107 | H | 0.67352 | 1.28103 | 0.01589 |
| H108 | H | 0.72372 | 1.25382 | 0.02196 |
| H109 | H | 0.78182 | 1.32826 | 0.33449 |
| H110 | H | 0.73187 | 1.35602 | 0.3265 |
| H111 | H | 0.72865 | 1.20035 | 0.18248 |
| H112 | H | 0.72026 | 1.1334 | 0.20491 |
| H113 | H | 0.77644 | 1.05412 | 0.21914 |
| H114 | H | 0.66224 | 1.07761 | 0.10753 |
| H115 | H | 0.63624 | 1.02033 | 0.09614 |
| H116 | H | 0.68689 | 1.05378 | 0.02196 |
| H117 | H | 0.66414 | 1.07724 | 0.32747 |
| H118 | H | 0.69252 | 1.05582 | 0.4063 |
| H119 | H | 0.64037 | 1.02005 | 0.34375 |
| H120 | H | 0.70697 | 0.99717 | 0.28949 |
| H121 | H | 0.70896 | 0.99941 | 0.13093 |
| H122 | H | 0.65808 | 0.97542 | 0.2056 |
| H123 | H | 0.87272 | 1.32706 | 0.39624 |
| H124 | H | 0.92237 | 1.40515 | 0.41019 |
| H125 | H | 0.89217 | 1.42054 | 0.05503 |
| H126 | H | 0.84388 | 1.34255 | 0.03979 |
| H127 | H | 0.99624 | 1.53952 | 0.50387 |
| H128 | H | 1.0527 | 1.6153 | 0.50314 |
| H129 | H | 1.06017 | 1.61233 | 0.12789 |
| H130 | H | 1.00274 | 1.53694 | 0.12774 |
| H131 | H | 1.10119 | 0.72492 | 0.31994 |
| H132 | H | 1.11824 | 0.71022 | 0.45283 |
| H133 | H | 1.06178 | 1.68664 | 0.41856 |
| H134 | H | 1.14139 | 0.69837 | 0.17983 |
| H135 | H | 1.1387 | 1.64801 | 0.21553 |
| H136 | H | 1.16554 | 1.693 | 0.31332 |
| N137 | N | 0.66667 | 1.33333 | 0.17218 |
| N138 | N | 1 | 1 | 0.27808 |

**Table S4. Cartesian coordinates for the optimized inclined AA stacking model**

Crystal system: Hexagonal

Space group: P1

International tables number: 1

a= 37.2 Å, b= 31.9 Å, c= 8.0 Å

α= 49.9^o^, β = 136.7^o^, γ = 119.8^o^

| C1 | C | 0.66746 | 0.38643 | 0.95308 |
| --- | --- | --- | --- | --- |
| C2 | C | 0.65388 | 0.3453 | 1.13301 |
| C3 | C | 0.62904 | 0.35884 | 1.16395 |
| C4 | C | 0.62144 | 0.41461 | 1.03406 |
| C5 | C | 0.64387 | 0.45592 | 0.89942 |
| C6 | C | 0.6651 | 0.44195 | 0.85225 |
| C7 | C | 0.59092 | 0.42961 | 1.02993 |
| C8 | C | 0.60871 | 0.46821 | 1.10783 |
| C9 | C | 0.56886 | 0.47322 | 1.07453 |
| C10 | C | 0.52623 | 0.43868 | 0.96911 |
| N11 | N | 0.5408 | 0.41204 | 0.9442 |
| C12 | C | 0.56858 | 0.50673 | 1.13058 |
| C13 | C | 0.52464 | 0.50201 | 1.10177 |
| C14 | C | 0.52527 | 0.53277 | 1.1765 |
| C15 | C | 0.61051 | 0.54473 | 1.21141 |
| C16 | C | 0.61427 | 0.57327 | 1.30427 |
| C17 | C | 0.57165 | 0.56403 | 1.29443 |
| C18 | C | 0.66169 | 0.61178 | 1.40303 |
| C19 | C | 0.6638 | 0.63537 | 1.53206 |
| C20 | C | 0.71474 | 0.58307 | 1.63209 |
| C21 | C | 0.6586 | 0.66166 | 1.14258 |
| C22 | C | 0.51287 | 0.37481 | 0.84712 |
| C23 | C | 0.48912 | 0.32498 | 0.95571 |
| C24 | C | 0.46369 | 0.28653 | 0.85888 |
| C25 | C | 0.46211 | 0.29739 | 0.65215 |
| C26 | C | 0.48638 | 0.34622 | 0.54693 |
| C27 | C | 0.51024 | 0.38471 | 0.63897 |
| N28 | N | 0.43679 | 0.26587 | 0.53169 |
| N29 | N | 0.39898 | 0.23009 | 0.52732 |
| C30 | C | 0.37005 | 0.20224 | 0.38345 |
| C31 | C | 0.32198 | 0.17724 | 0.33781 |
| C32 | C | 0.28788 | 0.15044 | 0.17504 |
| C33 | C | 0.30122 | 0.14784 | 0.0555 |
| C34 | C | 0.34956 | 0.17333 | 0.10084 |
| C35 | C | 0.38394 | 0.20004 | 0.26482 |
| N36 | N | 0.26753 | 0.12144 | -0.10109 |
| C37 | C | 0.25134 | 0.06405 | 0.01427 |
| C38 | C | 0.24589 | 0.15045 | -0.38754 |
| C39 | C | 0.64766 | 0.3318 | 0.79065 |
| C40 | C | 0.59318 | 0.32384 | 0.66051 |
| C41 | C | 0.56547 | 0.27356 | 0.65819 |
| C42 | C | 0.59026 | 0.23006 | 0.77267 |
| C43 | C | 0.64263 | 0.24134 | 0.86918 |
| C44 | C | 0.66993 | 0.29132 | 0.87054 |
| C45 | C | 0.56551 | 0.17659 | 0.80618 |
| C46 | C | 0.53089 | 0.15206 | 0.86765 |
| C47 | C | 0.51967 | 0.09766 | 0.92043 |
| C48 | C | 0.54658 | 0.08934 | 0.88336 |
| N49 | N | 0.57255 | 0.13836 | 0.80604 |
| C50 | C | 0.48838 | 0.05552 | 1.00106 |
| C51 | C | 0.46065 | 0.06428 | 1.03443 |
| C52 | C | 0.60161 | 0.1484 | 0.73955 |
| C53 | C | 0.6502 | 0.12402 | 0.94565 |
| C54 | C | 0.67977 | 0.13423 | 0.87378 |
| C55 | C | 0.66223 | 0.17035 | 0.59793 |
| C56 | C | 0.61457 | 0.19607 | 0.39517 |
| C57 | C | 0.58413 | 0.18495 | 0.46397 |
| N58 | N | 0.6894 | 0.18187 | 0.51905 |
| N59 | N | 0.72407 | 0.15081 | 0.65117 |
| C60 | C | 0.75266 | 0.16486 | 0.57539 |
| C61 | C | 0.76336 | 0.12396 | 0.59813 |
| C62 | C | 0.78593 | 0.1389 | 0.48117 |
| C63 | C | 0.79892 | 0.19493 | 0.34143 |
| C64 | C | 0.79292 | 0.23465 | 0.34687 |
| C65 | C | 0.77011 | 0.21986 | 0.46175 |
| N66 | N | 0.81513 | 0.21088 | 0.19848 |
| C67 | C | 0.79706 | 0.261 | -0.06546 |
| C68 | C | 0.85202 | 0.18 | 0.30851 |
| C69 | C | 0.48565 | 1.00207 | 1.05514 |
| C70 | C | 0.45212 | 0.95913 | 1.12135 |
| C71 | C | 0.42827 | 1.02234 | 1.10962 |
| C72 | C | 0.42392 | 0.97094 | 1.14216 |
| C73 | C | 0.39824 | 1.03006 | 1.15154 |
| C74 | C | 0.33868 | 1.03229 | 0.90293 |
| C75 | C | 0.41618 | 1.08387 | 1.17328 |
| C76 | C | 0.40453 | 0.98054 | 1.42305 |
| C77 | C | 0.01007 | 0.46034 | 1.10108 |
| C78 | C | 0.04585 | 0.44081 | 1.15001 |
| C79 | C | -0.02493 | 0.37511 | 0.96711 |
| C80 | C | 0.02781 | 0.39712 | 1.09361 |
| C81 | C | -0.04605 | 0.33227 | 0.8762 |
| C82 | C | -0.06951 | 0.27841 | 1.01969 |
| C83 | C | -0.0914 | 0.35658 | 0.55688 |
| C84 | C | -0.00492 | 0.3142 | 0.92932 |
| C85 | C | 0.72201 | 0.39903 | 0.86379 |
| C86 | C | 0.76746 | 0.41937 | 1.05252 |
| C87 | C | 0.81099 | 0.4437 | 1.05263 |
| C88 | C | 0.81218 | 0.44781 | 0.86903 |
| C89 | C | 0.76605 | 0.43046 | 0.6704 |
| C90 | C | 0.7231 | 0.40624 | 0.67369 |
| C91 | C | 0.85861 | 0.4641 | 0.89544 |
| C92 | C | 0.87353 | 0.43756 | 0.86864 |
| C93 | C | 0.92436 | 0.456 | 0.95478 |
| C94 | C | 0.93941 | 0.49714 | 1.01677 |
| N95 | N | 0.89816 | 0.50186 | 0.97322 |
| C96 | C | 0.9578 | 0.43731 | 0.9893 |
| C97 | C | 0.94126 | 0.39647 | 0.92002 |
| C98 | C | 0.89423 | 0.53608 | 1.00595 |
| C99 | C | 0.92575 | 0.53235 | 1.2674 |
| C100 | C | 0.91573 | 0.56433 | 1.29434 |
| C101 | C | 0.8731 | 0.59922 | 1.06175 |
| C102 | C | 0.84223 | 0.6032 | 0.80185 |
| C103 | C | 0.85307 | 0.57272 | 0.77301 |
| N104 | N | 0.85885 | 0.62919 | 1.06997 |
| N105 | N | 0.88569 | 0.64016 | 1.26762 |
| C106 | C | 0.86899 | 0.671 | 1.26068 |
| C107 | C | 0.90698 | 0.69527 | 1.43566 |
| C108 | C | 0.89238 | 0.72889 | 1.43409 |
| C109 | C | 0.83908 | 0.73893 | 1.25805 |
| C110 | C | 0.8009 | 0.71349 | 1.08596 |
| C111 | C | 0.81565 | 0.68021 | 1.08647 |
| N112 | N | 0.82501 | 0.77185 | 1.256 |
| C113 | C | 0.84984 | 0.82597 | 1.22696 |
| C114 | C | 0.78489 | 0.75436 | 1.28261 |
| C115 | C | 0.35088 | 0.59665 | 1.17551 |
| C116 | C | 0.31926 | 0.58635 | 0.95135 |
| C117 | C | 0.3414 | 0.56642 | 0.92843 |
| C118 | C | 0.39522 | 0.55567 | 1.12896 |
| C119 | C | 0.42477 | 0.5632 | 1.35754 |
| C120 | C | 0.40408 | 0.58459 | 1.37899 |
| C121 | C | 0.42057 | 0.53862 | 1.11105 |
| C122 | C | 0.40156 | 0.4979 | 1.03959 |
| C123 | C | 0.43738 | 0.49397 | 1.04057 |
| C124 | C | 0.47997 | 0.52994 | 1.13369 |
| N125 | N | 0.46708 | 0.55945 | 1.15238 |
| C126 | C | 0.43534 | 0.46413 | 0.95707 |
| C127 | C | 0.47995 | 0.46772 | 0.99179 |
| C128 | C | 0.47954 | 0.43918 | 0.90689 |
| C129 | C | 0.39026 | 0.43329 | 0.82832 |
| C130 | C | 0.38592 | 0.41031 | 0.70919 |
| C131 | C | 0.4315 | 0.41492 | 0.75103 |
| C132 | C | 0.33434 | 0.38308 | 0.54219 |
| C133 | C | 0.33661 | 0.35598 | 0.4409 |
| C134 | C | 0.29187 | 0.42694 | 0.2755 |
| C135 | C | 0.31581 | 0.33623 | 0.72298 |
| C136 | C | 0.49298 | 0.60746 | 1.16484 |
| C137 | C | 0.48904 | 0.65395 | 0.92801 |
| C138 | C | 0.51986 | 0.70264 | 0.93693 |
| C139 | C | 0.55531 | 0.70594 | 1.18442 |
| C140 | C | 0.55701 | 0.66107 | 1.41608 |
| C141 | C | 0.52813 | 0.6116 | 1.40991 |
| N142 | N | 0.59258 | 0.74691 | 1.22438 |
| N143 | N | 0.60453 | 0.78377 | 1.05721 |
| C144 | C | 0.64651 | 0.82021 | 1.10749 |
| C145 | C | 0.6494 | 0.86089 | 0.88727 |
| C146 | C | 0.6922 | 0.89988 | 0.92544 |
| C147 | C | 0.73384 | 0.89812 | 1.1873 |
| C148 | C | 0.73163 | 0.85614 | 1.41026 |
| C149 | C | 0.68818 | 0.81795 | 1.37049 |
| N150 | N | 0.77543 | 0.93578 | 1.22376 |
| C151 | C | 0.78638 | 0.97403 | 1.29832 |
| C152 | C | 0.8106 | 0.9359 | 1.20179 |
| C153 | C | 0.5164 | -0.00805 | 1.04011 |
| C154 | C | 0.54392 | 0.03602 | 0.93478 |
| C155 | C | 0.57186 | -0.03035 | 0.98212 |
| C156 | C | 0.56647 | 0.02395 | 0.88494 |
| C157 | C | 0.60074 | -0.04412 | 0.95483 |
| C158 | C | 0.61517 | 0.00654 | 0.75753 |
| C159 | C | 0.56754 | -0.08733 | 0.84444 |
| C160 | C | 0.65346 | -0.06873 | 1.24951 |
| C161 | C | 0.35563 | 0.66832 | 1.20903 |
| C162 | C | 0.36352 | 0.68015 | 1.37827 |
| C163 | C | 0.38776 | 0.73029 | 1.38702 |
| C164 | C | 0.40632 | 0.77007 | 1.23099 |
| C165 | C | 0.39979 | 0.75809 | 1.06756 |
| C166 | C | 0.37407 | 0.70906 | 1.05165 |
| C167 | C | 0.43147 | 0.82093 | 1.23182 |
| C168 | C | 0.47542 | 0.84206 | 1.2421 |
| C169 | C | 0.48453 | 0.89608 | 1.18558 |
| C170 | C | 0.44938 | 0.90622 | 1.16907 |
| N171 | N | 0.41706 | 0.85959 | 1.19527 |
| C172 | C | 0.51812 | 0.93734 | 1.12311 |
| C173 | C | 0.54885 | 0.92678 | 1.11294 |
| C174 | C | 0.37329 | 0.85542 | 1.15325 |
| C175 | C | 0.32717 | 0.87882 | 0.87952 |
| C176 | C | 0.2841 | 0.88309 | 0.83064 |
| C177 | C | 0.28638 | 0.86361 | 1.05479 |
| C178 | C | 0.3307 | 0.83564 | 1.32781 |
| C179 | C | 0.37408 | 0.8316 | 1.37776 |
| N180 | N | 0.24931 | 0.87239 | 1.02316 |
| N181 | N | 0.20952 | 0.90275 | 0.7873 |
| C182 | C | 0.17335 | 0.91752 | 0.7399 |
| C183 | C | 0.13336 | 0.95069 | 0.46171 |
| C184 | C | 0.0953 | 0.96914 | 0.39675 |
| C185 | C | 0.0962 | 0.9547 | 0.60808 |
| C186 | C | 0.13663 | 0.92159 | 0.88759 |
| C187 | C | 0.17471 | 0.9029 | 0.95312 |
| N188 | N | 0.05892 | 0.97334 | 0.53982 |
| C189 | C | 0.0079 | 0.9453 | 0.45616 |
| C190 | C | 0.06948 | 1.02331 | 0.54182 |
| C191 | C | 0.29456 | 0.59025 | 1.23607 |
| C192 | C | 0.25686 | 0.61785 | 1.12995 |
| C193 | C | 0.22002 | 0.58859 | 1.17993 |
| C194 | C | 0.21739 | 0.53011 | 1.32905 |
| C195 | C | 0.25524 | 0.50224 | 1.43748 |
| C196 | C | 0.29262 | 0.532 | 1.38963 |
| C197 | C | 0.1762 | 0.50438 | 1.35075 |
| C198 | C | 0.16297 | 0.52569 | 1.40527 |
| C199 | C | 0.11204 | 0.50706 | 1.31442 |
| C200 | C | 0.09727 | 0.46702 | 1.24769 |
| N201 | N | 0.13852 | 0.46465 | 1.28372 |
| C202 | C | 0.07794 | 0.52641 | 1.27177 |
| C203 | C | 0.09408 | 0.5691 | 1.32839 |
| C204 | C | 0.14134 | 0.43311 | 1.23479 |
| C205 | C | 0.11094 | 0.44298 | 0.96308 |
| C206 | C | 0.11559 | 0.41007 | 0.91937 |
| C207 | C | 0.15198 | 0.36826 | 1.14447 |
| C208 | C | 0.18292 | 0.35902 | 1.41515 |
| C209 | C | 0.17655 | 0.39015 | 1.46012 |
| N210 | N | 0.15747 | 0.33542 | 1.11263 |
| N211 | N | 0.11841 | 0.31935 | 0.92645 |
| C212 | C | 0.12003 | 0.28575 | 0.88368 |
| C213 | C | 0.07289 | 0.25715 | 0.75079 |
| C214 | C | 0.0714 | 0.22126 | 0.69937 |
| C215 | C | 0.11661 | 0.21393 | 0.77323 |
| C216 | C | 0.16401 | 0.2425 | 0.90942 |
| C217 | C | 0.16591 | 0.27757 | 0.96687 |
| N218 | N | 0.11364 | 0.18075 | 0.70944 |
| C219 | C | 0.13297 | 0.19975 | 0.5659 |
| C220 | C | 0.08974 | 0.12555 | 0.77641 |
| C221 | C | 1.02586 | 0.50331 | 1.16051 |
| C222 | C | 0.99049 | 0.52364 | 1.11016 |
| C223 | C | 1.0605 | 0.59239 | 1.27321 |
| C224 | C | 1.00824 | 0.5688 | 1.15706 |
| C225 | C | 1.07913 | 0.63838 | 1.34189 |
| C226 | C | 1.0786 | 0.61262 | 1.58536 |
| C227 | C | 1.13573 | 0.66711 | 1.43149 |
| C228 | C | 1.04123 | 0.68699 | 1.08725 |
| N229 | N | 0.33307 | 0.61879 | 1.20387 |
| N230 | N | 0.67911 | 0.37336 | 0.86355 |
| H231 | H | 0.65999 | 0.30491 | 1.23243 |
| H232 | H | 0.61626 | 0.32793 | 1.27911 |
| H233 | H | 0.64339 | 0.49634 | 0.82802 |
| H234 | H | 0.67784 | 0.47238 | 0.73781 |
| H235 | H | 0.64477 | 0.48858 | 1.17823 |
| H236 | H | 0.63963 | 0.55134 | 1.20737 |
| H237 | H | 0.57538 | 0.58011 | 1.38111 |
| H238 | H | 0.62939 | 0.6585 | 1.38049 |
| H239 | H | 0.69761 | 0.66413 | 1.591 |
| H240 | H | 0.66579 | 0.60246 | 1.71591 |
| H241 | H | 0.71913 | 0.56658 | 1.5633 |
| H242 | H | 0.71852 | 0.54962 | 1.8201 |
| H243 | H | 0.74781 | 0.61257 | 1.68938 |
| H244 | H | 0.62036 | 0.67979 | 0.97446 |
| H245 | H | 0.66524 | 0.64941 | 1.06686 |
| H246 | H | 0.68806 | 0.69405 | 1.19069 |
| H247 | H | 0.48962 | 0.31681 | 1.10309 |
| H248 | H | 0.44632 | 0.25098 | 0.93853 |
| H249 | H | 0.48593 | 0.35442 | 0.39908 |
| H250 | H | 0.52686 | 0.42003 | 0.55591 |
| H251 | H | 0.31126 | 0.17867 | 0.42109 |
| H252 | H | 0.25308 | 0.13267 | 0.14454 |
| H253 | H | 0.35988 | 0.17255 | 0.01418 |
| H254 | H | 0.41857 | 0.21825 | 0.29384 |
| H255 | H | 0.2096 | 0.06044 | -0.08775 |
| H256 | H | 0.27271 | 0.04471 | 0.23738 |
| H257 | H | 0.25798 | 0.04067 | -0.01749 |
| H258 | H | 0.20325 | 0.14977 | -0.51836 |
| H259 | H | 0.25564 | 0.13042 | -0.42071 |
| H260 | H | 0.26039 | 0.19362 | -0.45561 |
| H261 | H | 0.57394 | 0.35398 | 0.57807 |
| H262 | H | 0.52721 | 0.26854 | 0.57823 |
| H263 | H | 0.66342 | 0.21272 | 0.95371 |
| H264 | H | 0.70742 | 0.29695 | 0.94179 |
| H265 | H | 0.51741 | 0.17042 | 0.88043 |
| H266 | H | 0.46406 | 0.10239 | 1.00176 |
| H267 | H | 0.66373 | 0.09829 | 1.14715 |
| H268 | H | 0.71413 | 0.11512 | 1.02365 |
| H269 | H | 0.60133 | 0.22273 | 0.19527 |
| H270 | H | 0.54933 | 0.20347 | 0.31324 |
| H271 | H | 0.75307 | 0.08341 | 0.6905 |
| H272 | H | 0.79133 | 0.10833 | 0.49232 |
| H273 | H | 0.80419 | 0.27521 | 0.26198 |
| H274 | H | 0.76486 | 0.24992 | 0.45381 |
| H275 | H | 0.80344 | 0.26265 | -0.18273 |
| H276 | H | 0.81825 | 0.29705 | -0.03209 |
| H277 | H | 0.75511 | 0.26455 | -0.19567 |
| H278 | H | 0.83379 | 0.16395 | 0.17285 |
| H279 | H | 0.86698 | 0.14535 | 0.51688 |
| H280 | H | 0.88613 | 0.2067 | 0.32311 |
| H281 | H | 0.40009 | 0.94107 | 1.18895 |
| H282 | H | 0.32279 | 0.99343 | 0.89517 |
| H283 | H | 0.33267 | 1.06461 | 0.70862 |
| H284 | H | 0.31608 | 1.04095 | 0.91637 |
| H285 | H | 0.39598 | 1.08633 | 1.21853 |
| H286 | H | 0.4074 | 1.12077 | 0.97864 |
| H287 | H | 0.4583 | 1.08503 | 1.33749 |
| H288 | H | 0.39088 | 0.98968 | 1.4796 |
| H289 | H | 0.44558 | 0.97153 | 1.59087 |
| H290 | H | 0.38264 | 0.943 | 1.40449 |
| H291 | H | 0.05392 | 0.38065 | 1.14617 |
| H292 | H | -0.08685 | 0.24947 | 0.94285 |
| H293 | H | -0.1003 | 0.28756 | 0.98046 |
| H294 | H | -0.03939 | 0.25764 | 1.24317 |
| H295 | H | -0.10267 | 0.33121 | 0.47503 |
| H296 | H | -0.07874 | 0.39755 | 0.45077 |
| H297 | H | -0.12637 | 0.35965 | 0.50696 |
| H298 | H | -0.02182 | 0.28303 | 0.86576 |
| H299 | H | 0.02845 | 0.29647 | 1.14848 |
| H300 | H | 0.00958 | 0.34942 | 0.81348 |
| H301 | H | 0.77002 | 0.41567 | 1.19785 |
| H302 | H | 0.84282 | 0.45584 | 1.20007 |
| H303 | H | 0.76365 | 0.43406 | 0.52556 |
| H304 | H | 0.69221 | 0.39225 | 0.53699 |
| H305 | H | 0.85179 | 0.40738 | 0.81057 |
| H306 | H | 0.90321 | 0.3815 | 0.83175 |
| H307 | H | 0.95552 | 0.50575 | 1.43913 |
| H308 | H | 0.93904 | 0.56134 | 1.4843 |
| H309 | H | 0.81136 | 0.62817 | 0.63064 |
| H310 | H | 0.82973 | 0.57622 | 0.58178 |
| H311 | H | 0.94564 | 0.68894 | 1.563 |
| H312 | H | 0.92108 | 0.74606 | 1.5634 |
| H313 | H | 0.76195 | 0.71922 | 0.95794 |
| H314 | H | 0.78733 | 0.66241 | 0.96065 |
| H315 | H | 0.87556 | 0.83913 | 1.17706 |
| H316 | H | 0.8734 | 0.82635 | 1.42007 |
| H317 | H | 0.82042 | 0.85683 | 1.06037 |
| H318 | H | 0.79919 | 0.75956 | 1.44606 |
| H319 | H | 0.77256 | 0.71106 | 1.33606 |
| H320 | H | 0.7506 | 0.77894 | 1.08697 |
| H321 | H | 0.28027 | 0.5941 | 0.8025 |
| H322 | H | 0.31795 | 0.56022 | 0.76279 |
| H323 | H | 0.46217 | 0.55248 | 1.5165 |
| H324 | H | 0.42805 | 0.59131 | 1.54432 |
| H325 | H | 0.3669 | 0.47538 | 0.99186 |
| H326 | H | 0.35913 | 0.42879 | 0.81374 |
| H327 | H | 0.42928 | 0.4014 | 0.65868 |
| H328 | H | 0.29888 | 0.33582 | 0.3299 |
| H329 | H | 0.3657 | 0.32456 | 0.61676 |
| H330 | H | 0.34638 | 0.38668 | 0.2991 |
| H331 | H | 0.25446 | 0.40845 | 0.15144 |
| H332 | H | 0.30412 | 0.45987 | 0.14637 |
| H333 | H | 0.28457 | 0.44512 | 0.32601 |
| H334 | H | 0.34678 | 0.30752 | 0.91764 |
| H335 | H | 0.28092 | 0.31302 | 0.61774 |
| H336 | H | 0.30605 | 0.35217 | 0.77211 |
| H337 | H | 0.46531 | 0.65181 | 0.75028 |
| H338 | H | 0.51769 | 0.73476 | 0.76572 |
| H339 | H | 0.58161 | 0.66289 | 1.59558 |
| H340 | H | 0.53549 | 0.57801 | 1.59048 |
| H341 | H | 0.62024 | 0.86244 | 0.69739 |
| H342 | H | 0.6929 | 0.92973 | 0.76109 |
| H343 | H | 0.76153 | 0.85357 | 1.60247 |
| H344 | H | 0.68731 | 0.78853 | 1.53452 |
| H345 | H | 0.81498 | 0.95699 | 1.52363 |
| H346 | H | 0.75108 | 0.98205 | 1.22965 |
| H347 | H | 0.80255 | 1.01369 | 1.20521 |
| H348 | H | 0.80881 | 0.97465 | 1.02778 |
| H349 | H | 0.80116 | 0.90199 | 1.16941 |
| H350 | H | 0.85079 | 0.9315 | 1.39264 |
| H351 | H | 0.57889 | 0.05529 | 0.77201 |
| H352 | H | 0.58019 | 0.02807 | 0.55169 |
| H353 | H | 0.63341 | -0.00621 | 0.73548 |
| H354 | H | 0.64299 | 0.03568 | 0.83815 |
| H355 | H | 0.52676 | -0.07648 | 0.67532 |
| H356 | H | 0.56854 | -0.12903 | 1.00528 |
| H357 | H | 0.58145 | -0.08914 | 0.76745 |
| H358 | H | 0.67673 | -0.07736 | 1.24109 |
| H359 | H | 0.6766 | -0.0396 | 1.33161 |
| H360 | H | 0.64714 | -0.10724 | 1.39613 |
| H361 | H | 0.35238 | 0.65177 | 1.502 |
| H362 | H | 0.39294 | 0.73654 | 1.5139 |
| H363 | H | 0.41352 | 0.78537 | 0.95536 |
| H364 | H | 0.36932 | 0.70335 | 0.92406 |
| H365 | H | 0.49524 | 0.82215 | 1.26924 |
| H366 | H | 0.55298 | 0.88703 | 1.19243 |
| H367 | H | 0.32531 | 0.89441 | 0.71462 |
| H368 | H | 0.25164 | 0.90174 | 0.63039 |
| H369 | H | 0.33198 | 0.81964 | 1.49103 |
| H370 | H | 0.40676 | 0.81302 | 1.57668 |
| H371 | H | 0.13175 | 0.96185 | 0.30421 |
| H372 | H | 0.06681 | 0.99338 | 0.19366 |
| H373 | H | 0.13834 | 0.91089 | 1.04435 |
| H374 | H | 0.2034 | 0.87887 | 1.15636 |
| H375 | H | 0.0009 | 0.93403 | 0.59198 |
| H376 | H | 0.00458 | 0.90739 | 0.46759 |
| H377 | H | -0.02265 | 0.97204 | 0.2427 |
| H378 | H | 0.06554 | 1.01492 | 0.68375 |
| H379 | H | 0.04154 | 1.0539 | 0.33372 |
| H380 | H | 0.10893 | 1.04167 | 0.60683 |
| H381 | H | 0.25567 | 0.65992 | 1.01347 |
| H382 | H | 0.19392 | 0.61131 | 1.09734 |
| H383 | H | 0.25684 | 0.46049 | 1.55493 |
| H384 | H | 0.31943 | 0.51075 | 1.47198 |
| H385 | H | 0.18537 | 0.55408 | 1.47529 |
| H386 | H | 0.13176 | 0.58463 | 1.41126 |
| H387 | H | 0.08508 | 0.47373 | 0.79725 |
| H388 | H | 0.09241 | 0.41664 | 0.7229 |
| H389 | H | 0.20957 | 0.32898 | 1.58149 |
| H390 | H | 0.1986 | 0.38211 | 1.65857 |
| H391 | H | 0.03938 | 0.26198 | 0.69238 |
| H392 | H | 0.03661 | 0.20093 | 0.60302 |
| H393 | H | 0.19766 | 0.23764 | 0.96932 |
| H394 | H | 0.20071 | 0.2974 | 1.06699 |
| H395 | H | 0.14928 | 0.2421 | 0.51651 |
| H396 | H | 0.10115 | 0.19809 | 0.37071 |
| H397 | H | 0.16372 | 0.17369 | 0.69296 |
| H398 | H | 0.08029 | 0.11209 | 0.90621 |
| H399 | H | 0.11625 | 0.09599 | 0.89046 |
| H400 | H | 0.05351 | 0.12335 | 0.58625 |
| H401 | H | 0.98259 | 0.58591 | 1.10438 |
| H402 | H | 1.03915 | 0.59777 | 1.52652 |
| H403 | H | 1.09272 | 0.64257 | 1.65066 |
| H404 | H | 1.1036 | 0.57757 | 1.76361 |
| H405 | H | 1.13758 | 0.68789 | 1.26332 |
| H406 | H | 1.16526 | 0.63648 | 1.61052 |
| H407 | H | 1.14616 | 0.69754 | 1.48592 |
| H408 | H | 1.03766 | 0.70295 | 0.90725 |
| H409 | H | 1.05543 | 0.72099 | 1.12894 |
| H410 | H | 1.00231 | 0.674 | 1.03432 |
